# Bimodal Evans–Polanyi Relationships in Hydrogen Atom Transfer from C(sp^3^)–H Bonds to the Cumyloxyl Radical. A Combined Time-Resolved Kinetic and Computational Study

**DOI:** 10.1021/jacs.1c05566

**Published:** 2021-07-26

**Authors:** Michela Salamone, Marco Galeotti, Eduardo Romero-Montalvo, Jeffrey A. van Santen, Benjamin D. Groff, James M. Mayer, Gino A. DiLabio, Massimo Bietti

**Affiliations:** † Dipartimento di Scienze e Tecnologie Chimiche, Università “Tor Vergata”, Via della Ricerca Scientifica, 1, I-00133 Rome, Italy; ‡ Department of Chemistry, The University of British Columbia, 3247 University Way, Kelowna, British Columbia, Canada, V1V 1V7; § Department of Chemistry, Yale University, 225 Prospect Street, New Haven, Connecticut 06520-8107, United States

## Abstract

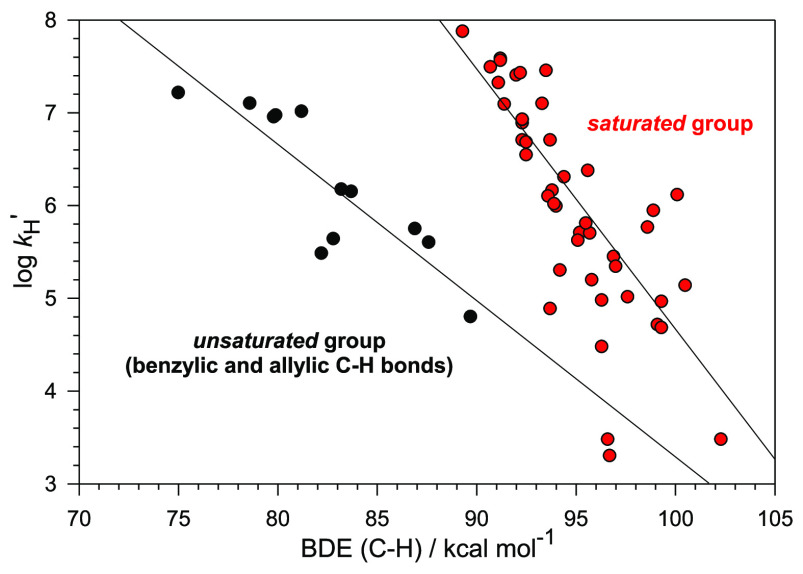

The applicability of the Evans–Polanyi (EP) relationship to HAT reactions from C(sp^3^)–H bonds to the cumyloxyl radical (CumO^•^) has been investigated. A consistent set of rate constants, *k*_H_, for HAT from the C–H bonds of 56 substrates to CumO^•^, spanning a range of more than 4 orders of magnitude, has been measured under identical experimental conditions. A corresponding set of consistent gas-phase C–H bond dissociation enthalpies (BDEs) spanning 27 kcal mol^–1^ has been calculated using the (RO)CBS-QB3 method. The log *k*_H_^′^ vs C–H BDE plot shows two distinct EP relationships, one for substrates bearing benzylic and allylic C–H bonds (*unsaturated* group) and the other one, with a steeper slope, for saturated hydrocarbons, alcohols, ethers, diols, amines, and carbamates (*saturated* group), in line with the bimodal behavior observed previously in theoretical studies of reactions promoted by other HAT reagents. The parallel use of BDFEs instead of BDEs allows the transformation of this correlation into a linear free energy relationship, analyzed within the framework of the Marcus theory. The Δ*G*^⧧^_HAT_ vs Δ*G*°_HAT_ plot shows again distinct behaviors for the two groups. A good fit to the Marcus equation is observed only for the saturated group, with λ = 58 kcal mol^–1^, indicating that with the unsaturated group λ must increase with increasing driving force. Taken together these results provide a qualitative connection between Bernasconi’s principle of nonperfect synchronization and Marcus theory and suggest that the observed bimodal behavior is a general feature in the reactions of oxygen-based HAT reagents with C(sp^3^)–H donors.

## Introduction

The Evans–Polanyi (EP) relationship (also known as the Bell–Evans–Polanyi relationship) correlates reaction rate constants (or other activation parameters) with bond dissociation enthalpies (BDEs).^1,2^ The relationship is often employed as a mechanistic tool in C–H bond oxidations promoted by radical and radical-like species, where the observation of a correlation is taken as evidence for a C–H bond cleavage step that occurs through hydrogen atom transfer (HAT).^3−7^ From the correlation obtained for the reaction of a given HAT reagent with a series of substrates or, alternatively, of a given substrate with a series of HAT reagents,^3,8^ it is possible to predict rate constants for the corresponding reactions of additional substrates,^9^ as well as derive substrate BDEs or the BDE of the new bond formed by the HAT reagent following abstraction.^10,11^

In 1982, Tedder discussed the factors governing reactivity and selectivity in atom transfer reactions.^12^ He highlighted the relative importance of the strengths of both the bond being broken and the bond being formed and of polar and steric effects in these processes. Tedder pointed out that in atom transfer reactions by a given radical the EP relationship will hold when the reaction is accompanied by a small change in polarity on going from the reactants to the associated transition state.^13^ More recently, one of us found that the empirical extra-thermodynamic relationship defined by EP holds quite well for HAT reactions over a wide range of driving force, when comparing similar radicals and similar substrates, for example for HAT from C–H bonds to oxygen-centered radicals.^3a^ This work also highlighted the limitations imposed by the difficulty in compiling a consistent set of C–H bond strengths. The same limitation, together with the importance of extending the correlation over a sufficiently broad range of C–H bond strengths, was also evidenced in a recent work by Jackson and co-workers in the case of HAT reactions promoted by high-valent metal oxo species.^14^

Tanko and co-workers examined the kinetics for HAT from the C(sp^3^)–H bonds of a series of 22 amine, hydrocarbon, alcohol, and ether substrates to the *tert*-butoxyl radical ((CH_3_)_3_CO^•^, *t*BuO^•^).^15^ No simple relationship was observed between the log of the HAT rate constant *k*_H_ normalized by the number of equivalent abstractable hydrogen atoms *n* (i.e., log *k*_H_^′^ where *k*_H_^′^ = *k*_H_/*n*) and the pertinent C–H BDE taken from the literature or, when not available, calculated by density-functional theory (DFT). Tanko observed very similar *k*_H_^′^ values for the reaction of *t*BuO^•^ with triallylamine and triethylamine, and with toluene and cyclohexane, despite the fact that both substrate couples have C(sp^3^)–H bonds that differ in strength by 8–10 kcal mol^–1^. An apparent curvature in the log *k*_H_^′^ vs C–H BDE plot was observed. For substrates with C–H BDEs greater than 92 kcal mol^–1^, log *k*_H_^′^ decreased with increasing bond strength, whereas for substrates with C–H BDEs smaller than 92 kcal mol^–1^, log *k*_H_^′^ appeared to be independent of the C–H BDE and to level off at a value of about 6.6. The analysis of the Arrhenius parameters for the different reactions led to the proposal that, at room temperature, most HAT reactions from C–H bonds to *t*BuO^•^ are entropy controlled.

In recent work by Houk and co-workers, a bimodal EP relationship was found through DFT modeling for the C(sp^3^)–H bond oxidation of a series of 18 substrates promoted by dimethyldioxirane (DMDO).^16^ The authors proposed that these reactions proceed through a rate-determining HAT step followed by fast OH rebound. By plotting Δ*H*^⧧^ vs C–H BDE, correlations with different slopes (∂Δ*H*^⧧^/∂Δ*H*°) were observed for the oxidation of aliphatic C–H bonds (*saturated* group) and for those of benzylic, allylic, and α to C=O or C≡N C–H bonds (*unsaturated* group). Within the *saturated* group of C–H oxidations by DMDO, ∂Δ*H*^⧧^/∂Δ*H*° = 0.91, indicating that the energies of the transition states and of the intermediate carbon-centered radicals are influenced to almost the same extent by substrate structure. In the *unsaturated* group, however, the transition state energies reflect little of the resonance stabilization of the allylic or benzylic radical products of HAT (∂Δ*H*^⧧^/∂Δ*H*° = 0.35). The differences were rationalized on the basis of Bernasconi’s principle of nonperfect synchronization (PNS),^17,18^ that the *unsaturated* reactions are characterized by an “imbalanced transition state”. Support for this picture was obtained by calculating, within the *unsaturated* group, the bond length of the C(sp^2^)–C(sp^3^) bond connecting the C(sp^3^)–H bond to be cleaved to the π-system both in the transition state and in the intermediate radical, which was taken as a measure of developing resonance stabilization. For all members of the group a significantly shorter bond length was observed in the radical as compared to the transition state, in line with increased resonance stabilization along the reaction coordinate. In this study, in contrast with Tedder’s indication,^12^ no significant deviation from the correlation was observed for substrates characterized by the presence of C–H α to polar groups.

Interestingly, a reexamination of Tanko’s data (Supporting Information (SI), Figures S1 and S2)^15^ shows that the log *k*_H_^′^ vs C–H BDE plot can be roughly divided into separate *saturated* and *unsaturated* groups, analogous to the groupings proposed by Houk.^16^ Specifically, substrates where HAT occurs from aliphatic and α to heteroatom C–H bonds appear to fall along one correlation line, while those for which HAT occurs from benzylic and allylic C–H bonds fall along a different line with a significantly different slope (Figure S2).

The PNS was also invoked by Korzekwa and co-workers to account for the results obtained in a computational study of HAT from the C(sp^3^)–H bonds in a series of 20 substrates to *p*-nitrosophenoxyl radical, taken as a model for the first step in cytochrome P450-mediated hydroxylation reactions.^19^ A modest correlation was observed when plotting activation enthalpies Δ*H*^⧧^ vs reaction enthalpies Δ*H*_R_, where conjugated systems (the *unsaturated* group) were observed to lie above the correlation line, suggesting that with these substrates resonance stabilization of the product radical provides only limited stabilization to the corresponding transition state. The calculated intrinsic barriers associated with the conjugated substrates were observed to be higher than those associated with the unconjugated counterparts, in line with Bernasconi’s PNS stating that a product stabilizing factor that develops late along the reaction coordinate always increases the intrinsic barrier.^17^ An excellent correlation was obtained by correcting the disproportionate product stabilization by means of a resonance parameter.^19^ A valence bond approach that predicts such bimodal behavior based on a delocalization penalty was also described.^20,21^

In keeping with the common mechanistic rationalization in terms of the PNS that was provided by the Houk^16^ and Korzekwa^19^ studies, it is important to point out that this principle and its implication of imbalanced or asynchronous transition states is currently being applied to proton-coupled electron transfer (PCET) processes involving C–H,^22−24^ O–H, and N–H bonds.^25^ Such processes are increasingly being discussed using a Marcus-theory-type approach, which is based on free energies rather than the enthalpies more traditional for organic HAT reactions. The PNS was primarily discussed in terms of free energies as well.

A critical examination of the studies referenced above^3−7^ shows that the reported correlations are based on a relatively small number of hydrogen atom donor substrates, typically between four and nine. The substrates subjected to study typically contained benzylic or allylic C–H bonds, with limited or no inclusion of substrates bearing unactivated aliphatic C–H bonds (cyclohexane and, to a lesser extent, 2,3-dimethylbutane and cyclooctane). These features prevent a thorough analysis of the experimental data and a possible assessment of the generality of the bimodal behavior observed for HAT from the C(sp^3^)–H bonds of the *saturated* and *unsaturated* substrate groups discussed above.^15,16,19^

In view of the important role played by HAT reactions from C(sp^3^)–H bonds in both chemical and biological processes,^26−28^ we sought to develop a deeper understanding of the scope and applicability of the EP relationship to this class of reactions. In keeping with our ongoing interest in HAT reactions involving oxygen-centered radicals,^29^ we compiled from our previous work and carried out additional detailed time-resolved kinetic studies in acetonitrile solution of the reactions of the cumyloxyl radical (PhC(CH_3_)_2_O^•^, CumO^•^) with an extended series of hydrogen atom donor substrates (structures **1**–**56** displayed in Table 1 and Table 2). The substrates were selected to cover the broadest possible variety of C(sp^3^)–H bonds (unactivated aliphatic, benzylic and allylic, α to heteroatom (O, N), formylic, and α to an electron-withdrawing functional group) and associated bond strengths. CumO^•^ is a well-established HAT reagent and an ideal radical probe for the direct measurement of HAT rate constants by nanosecond laser flash photolysis (LFP) over a broad reactivity range.^30^

**Table 1:**
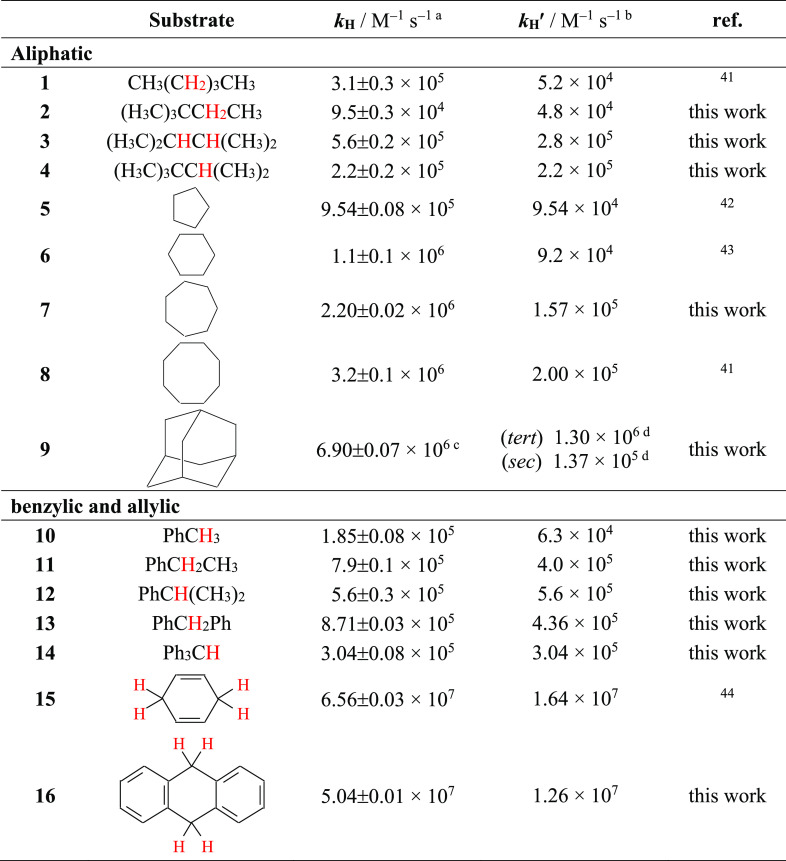
Second-Order Rate Constants (*k*_H_) for Reaction of the Cumyloxyl Radical (CumO^•^) with Hydrocarbon Substrates

a Measured in Ar- or N_2_-saturated MeCN solution at *T* = 25 °C by 355 nm LFP, [dicumyl peroxide] = 1.0 M. *k*_H_ values were determined from the slope of the *k*_obs_ vs [substrate] plots, where in turn *k*_obs_ values were measured following the decay of the CumO^•^ visible absorption band at 490 nm. Average of at least two determinations.

b *k*_H_^′^ = *k*_H_/*n*, where *n* represents the number of equivalent abstractable hydrogen atoms.

c Measured in isooctane solution.

d Derived from the measured *k*_H_ value, taking into account the product distribution observed after reaction of CumO^•^ with adamantane in oxygen-saturated isooctane solution (see text).

**Table 2:**
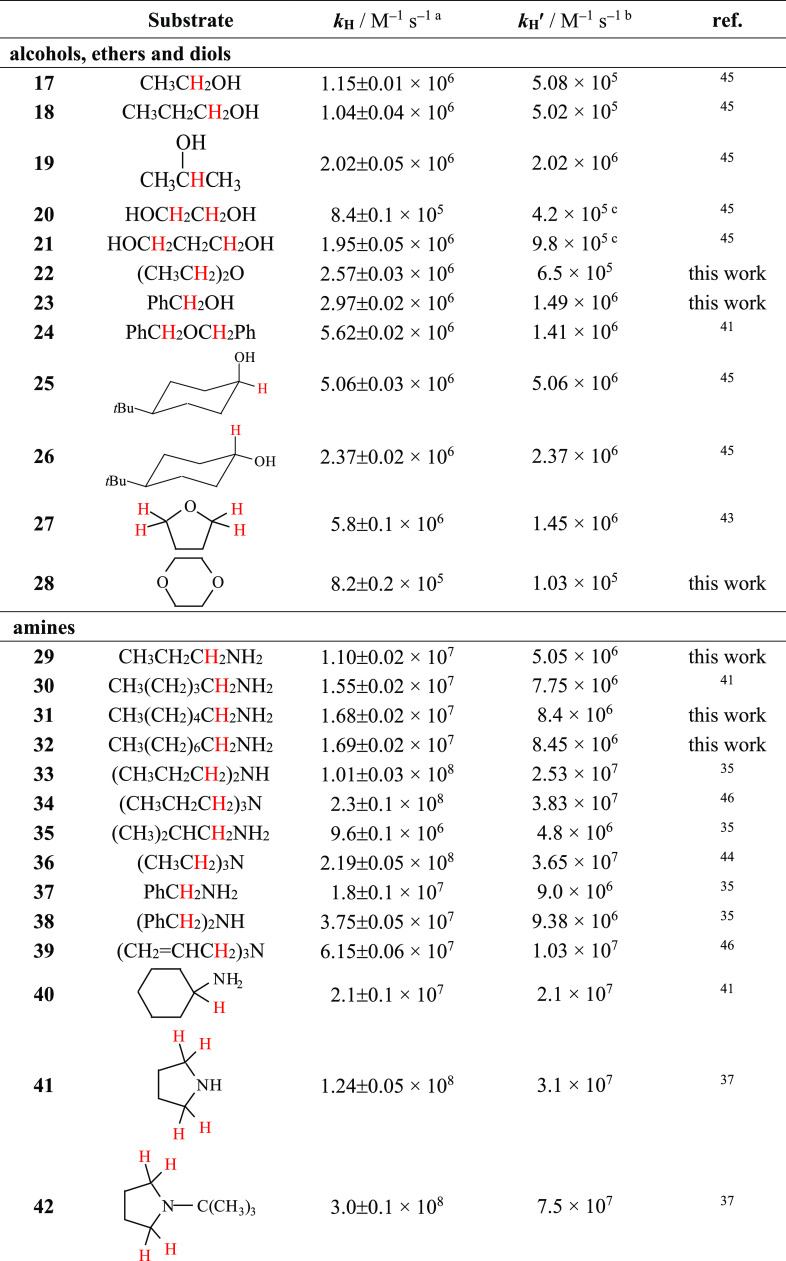

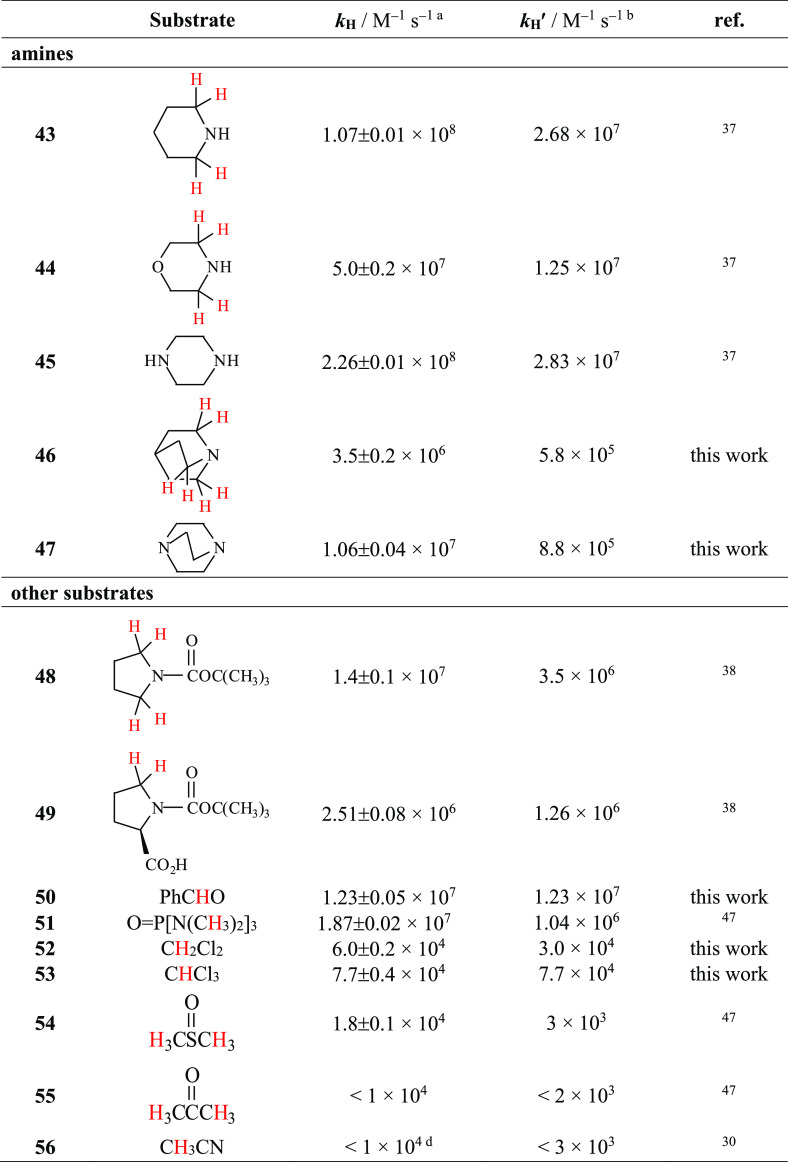
Second-Order Rate Constants (*k*_H_) for Reaction of the Cumyloxyl Radical (CumO^•^) with Different Substrates

a Measured in Ar- or N_2_-saturated MeCN solution at *T* = 25 °C by 355 nm LFP, [dicumyl peroxide] = 1.0 M. *k*_H_ values were determined from the slope of the *k*_obs_ vs [substrate] plots, where in turn *k*_obs_ values were measured following the decay of the CumO^•^ visible absorption band at 490 nm. Average of at least two determinations.

b *k*_H_^′^ = *k*_H_/*n*, where *n* represents the number of equivalent abstractable hydrogen atoms.

c Calculated considering *n* = 2 based on the difference between the C–H BDEs of the two methylene groups in an intramolecular hydrogen-bonded structure (see text).

d Measured in isooctane solution.^41−46^

In the present work, C–H BDE values were initially taken from Luo’s most recent compilation of chemical bond energies.^31^ However, because of the lack of C–H BDEs for some of the substrates used in this study, the large discrepancy between the available values for some substrates, and the large difference between the available values for some structurally related substrates, we decided instead to calculate the gas-phase C–H BDEs for all substrates. We used the (RO)CBS-QB3 method and, where feasible, also used W1BD for benchmarking purposes. Below we compare these results to the data taken from Luo’s compilation. Because one of us has advocated the use of bond dissociation *free energies* (BDFEs) instead of BDEs,^3b^ we have also calculated the gas-phase C–H BDFEs for all substrates.

Our approach provides a consistent set of experimental *k*_H_ values for HAT from the most activated C–H bonds of substrates **1**–**56** to CumO^•^, measured under identical conditions, and a corresponding set of consistent C–H BDEs and BDFEs. These data allow a detailed exploration of the applicability of the EP relationship to HAT reactions from the C–H bonds of an extensive set of hydrogen atom donor substrates, with *k*_H_ values spanning a range of more than 4 orders of magnitude and C–H BDEs spanning a range of 27 kcal mol^–1^. Moreover, the parallel use of BDFEs instead of BDEs allows the transformation of this correlation into a linear free energy relationship that can be conveniently analyzed within the framework of the Marcus theory. We also briefly connect this work to prior analyses using valence bond state correlation diagrams (VBSCDs).

## Results

### Time-Resolved Kinetic Studies

CumO^•^ was generated by 355 nm LFP of nitrogen or argon-saturated acetonitrile or 2,2,4-trimethylpentane (isooctane) solutions (*T* = 25 °C) containing 1.0 M dicumyl peroxide (eq 1).
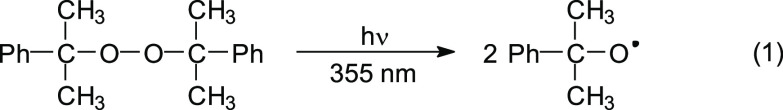
1

In aprotic solvents, CumO^•^ is characterized by a broad absorption band in the visible region of the spectrum centered at 485 nm.^30^ Under these conditions, CumO^•^ decays mainly by C–CH_3_ β-scission. The reactions of CumO^•^ with the different hydrogen atom donor substrates were studied using the LFP technique. Care was taken to select substrates containing equivalent aliphatic C–H bonds or, in those containing nonequivalent aliphatic C–H bonds, substrates from which HAT to CumO^•^ predominantly or almost exclusively occurs from a single C–H site or type of site. Previous studies clearly show that *tert*-alkoxyl radicals display very low reactivity toward the C–H bonds of unactivated methyl groups (*k*_H_ < 1 × 10^4^ M^–1^ s^–1^)^32,33^ and are essentially unreactive toward alkenyl and aryl C(sp^2^)–H bonds.^33,34^ HAT from primary and secondary amines and alcohols is known to occur predominantly at C–H bonds α to the heteroatom rather than from the N–H and O–H bonds, respectively.^35,36^

The kinetic measurements were carried out by LFP in acetonitrile by following the decay of the CumO^•^ visible absorption band as a function of substrate concentration. Because of the poor solubility of adamantane (**9**) in acetonitrile, the reaction of CumO^•^ with this substrate was studied in isooctane. The observed rate constants (*k*_obs_) gave excellent linear relationships when plotted against substrate concentration (typical *r*^2^ values >0.99), with intercepts close to that for CumO^•^ β-scission. The second-order rate constants for HAT (*k*_H_) were obtained from the slopes of these plots (see the SI). The *k*_H_ values thus obtained for the reaction of CumO^•^ with alkanes and cycloalkanes (substrates **1**–**9**) and with benzylic and allylic hydrocarbons (substrates **10**–**16**) are collected in Table 1. Table 2 contains the *k*_H_ values measured for reaction of CumO^•^ with alcohols, ethers, diols (substrates **17**–**28**), amines (substrates **29**–**47**), and with *N*-*tert*-butoxycarbonylpyrrolidine (*N*-Boc-pyrrolidine, **48**), *N*-Boc-l-proline (**49**), benzaldehyde (**50**), hexamethylphosphoric acid triamide (HMPA, **51**), dichloromethane (**52**), chloroform (**53**), dimethyl sulfoxide (DMSO, **54**), acetone (**55**), and acetonitrile (**56**). In both tables, the C–H bonds undergoing HAT to CumO^•^ are highlighted in red. All of the sp^3^ C–H bonds in substrates **5**–**8**, **10**, **13**–**16**, **23**, **24**, **28**, **37**–**39**, **45**, **47**, and **51**–**56** are equivalent. HAT from tetrahydrofuran (**27**) to CumO^•^ predominantly occurs from four equivalent α-C–H bonds. HAT from cyclohexyl amine (**40**), from cyclic and bicyclic amines (**41**–**44**, **46**), and from *N*-Boc-pyrrolidine (**48**) to CumO^•^ occurs almost exclusively from the C–H bonds that are α to the nitrogen atom.^35,37,38^ HAT from *N*-Boc-l-proline (**49**) has been shown to occur selectively from the δ-C–H bonds.^38^ HAT from benzaldehyde (**50**) occurs selectively from the formylic C–H bond.^39^ Also included in Table 1 and 2 are the *k*_H_^′^ values, obtained by dividing the measured *k*_H_ value by the number of equivalent abstractable hydrogen atoms, *n* (*k*_H_^′^ = *k*_H_/*n*).

For adamantane (**9**), the partial rate constants for HAT from the secondary and tertiary C–H bonds were derived from the product distribution observed after reaction of CumO^•^ with this substrate in oxygen-saturated isooctane solution (for details see the SI). Under these experimental conditions, the reaction of adamantane led to the formation of products derived from secondary C–H bond oxidation (2-adamantanone) and tertiary C–H bond oxidation (1-adamantanol and 1,3-adamantanediol) in a 0.31 ratio. This result is in excellent agreement with the product ratio determined previously for HAT from adamantane to *t*BuO^•^ (0.28)^40^ and is in line with the almost identical HAT reactivity displayed by these two *tert*-alkoxyl radicals.^35^ By taking into account the measured *k*_H_ value for HAT from adamantane to CumO^•^, *k*_H_ = 6.90 × 10^6^ M^–1^ s^–1^, and the number of secondary and tertiary C–H bonds, the partial rate constants for HAT from the secondary and tertiary C–H bonds of adamantane can be obtained as *k*_H_^′^(sec) = 1.37 × 10^5^ M^–1^ s^–1^ and *k*_H_^′^(tert) = 1.30 × 10^6^ M^–1^ s^–1^.

The *k*_H_ values for HAT to CumO^•^ displayed in Tables 1 and 2 span a range of more than 4 orders of magnitude. On the low end of the range are substrates such as acetone (**55**) and acetonitrile (**56**), which contain electron-poor C–H bonds that are strongly deactivated toward HAT to the electrophilic CumO^•^.^30,48^ With these two substrates, we could only determine an upper limit to *k*_H_ (<1 × 10^4^ M^–1^ s^–1^). On the high end of the range (*k*_H_ ≈ (1–3) × 10^8^ M^–1^ s^–1^) are substrates such as tertiary alkylamines, which contain electron-rich and strongly activated α-C–H bonds.^15,35,37,49^

**Figure 1:**
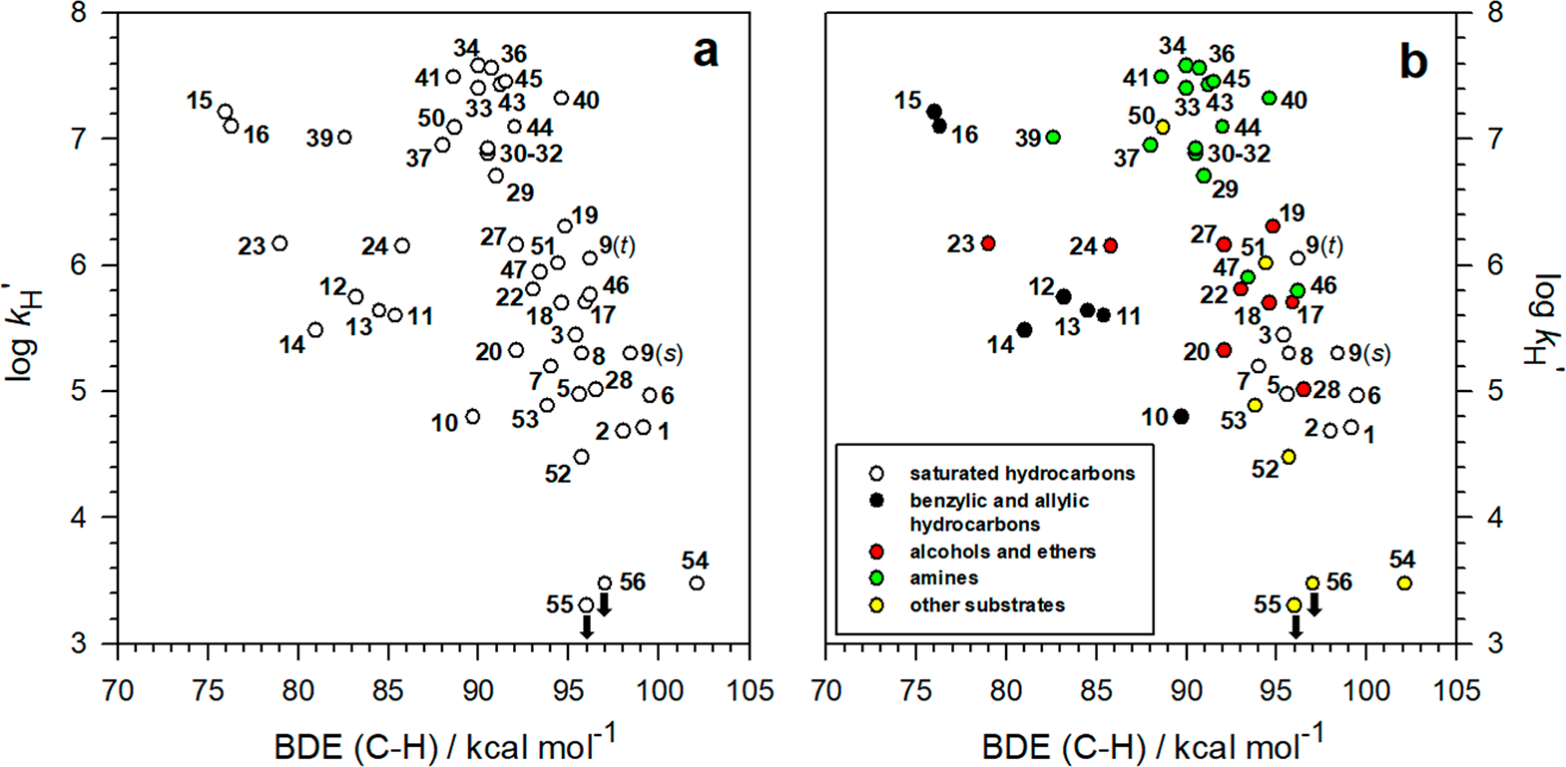
(a) Plot of log *k*_H_^′^ vs C–H BDE for reaction of the cumyloxyl radical (CumO^•^) with substrates **1**–**56**, the structures for which are displayed in Tables 1 and 2. The BDE values are mostly though not entirely from Luo; see text. Substrates **4**, **21**, **25**, **26**, **35**, **38**, **42**, **48**, and **49** have been omitted from the plot (see text). The plotted log *k*_H_^′^ values for **55** and **56** are upper limits. (b) Same plot with kinetic data grouped on the basis of substrate type.

Figure 1 shows the plot of log *k*_H_^′^ vs C–H BDE for reaction of CumO^•^ with most of the substrates displayed in Tables 1 and 2. BDE values are those recommended in Luo’s compilation.^31^ For substrates where Luo provides more than one value without a recommendation, the BDE was taken as the average of the tabulated values. Luo’s compilation does not contain BDE values for the tertiary C–H bond of 2,2,3-trimethylbutane (**4**) or the C–H bonds α to the OH, NH_2_, or NH groups of 1,3-propanediol (**21**), *cis*-4-*tert*-butylcyclohexanol (**25**), *trans*-4-*tert*-butylcyclohexanol (**26**), isobutylamine (**35**) dibenzylamine (**38**), *N*-*tert*-butylpyrrolidine (**42**), *N*-Boc-pyrrolidine (**48**), and *N*-Boc-proline (**49**), and these substrates were omitted from the plot. The C–H BDE values for HMPA (**51**) and DMSO (**54**), 94.4 and 102.1 kcal mol^–1^, respectively, were taken from our recent work in which we discussed the large discrepancy between Luo’s tabulated value for DMSO of 94.0 kcal mol^–1^ and our computed value.^47^ The BDEs for the α-C–H bonds of hexylamine (**31**) and octylamine (**32**) are assumed to be identical to the tabulated value for pentylamine (**30**).^31^ The BDE for the α-C–H bonds of dipropylamine (**33**) is assumed to be identical to the tabulated value for tripropylamine (**34**).^31^ All the data employed for the log *k*_H_^′^ vs C–H BDE plot displayed in Figure 1 are collected in Table S1 in the SI. For comparison, the O–H BDE of 2-phenylpropan-2-ol (cumyl alcohol) is given by Luo as 104.7 ± 0.2 kcal mol^–1^,^31^ essentially at the right axis of the plots.

The overall view of Figure 1a suggests that there is not a simple relationship between log *k*_H_^′^ and C–H BDE. However, grouping the kinetic data based on substrate type, i.e., benzylic/allylic hydrocarbons (black circles), saturated hydrocarbons (white circles), alcohols and ethers (red circles), amines (green circles), and other substrates (yellow circles), reveals two broad relationships (Figure 1b) that are similar to those observed by Tanko and co-workers for HAT reactions involving *t*BuO^•^ with their smaller set of substrates (SI, Figure S2).^15^ However, unlike the results of Tanko, the plot displayed in Figure 1b shows no leveling off of log *k*_H_^′^ at around 6.6.

Analysis of the data points displayed in Figure 1b shows that benzyl alcohol (**23**) and dibenzyl ether (**24**) do not follow the same general trend established by the alcohol and ether substrates, nor does triallylamine (**39**) fit the general trend associated with the amine substrates. Given their very weak C–H bonds, these substrates would have been expected to react with *much* higher rate constants. HAT from **23**, **24**, and **39** to CumO^•^ occurs selectively from the benzylic and allylic C–H bonds, and the corresponding log *k*_H_^′^ values appear to fit fairly well to the benzylic/allylic correlation (black circles).

### Computations of a Consistent Set of BDEs and BDFEs

One would reasonably expect very similar BDEs for the benzylic C–H bonds of benzyl alcohol (**23**) and dibenzyl ether (**24**), but quite surprisingly the tabulated values differ by 6.8 kcal mol^–1^, viz., 79.0 and 85.8 kcal mol^–1^,^31^ respectively. From this perspective, the tabulated BDE of 88.0 kcal mol^–1^ for the benzylic C–H bonds of benzylamine (**37**) seems to be too high.^31^ These apparent discrepancies in the BDEs, along with the absence of BDE values for the C–H bonds of some of the substrates listed in Tables 1 and 2, prompted us to use computational methods to generate a consistent set of gas-phase C–H BDEs for substrates **1**–**56**. We calculated the relevant C–H BDEs using the (RO)CBS-QB3 approach and present these data in Table 3 (column 4). For comparison, Luo’s tabulated values are shown in column 3 of Table 3. According to the benchmarking data we present in the SI, the (RO)CBS-QB3 approach predicts BDEs that are in excellent agreement with the BDEs we computed for 22 out of the 56 substrates using the high-level W1BD approach (mean absolute error, MAE = 0.26 kcal mol^–1^). Additional analysis of the calculated BDEs is provided in the SI.

**Table 3:**
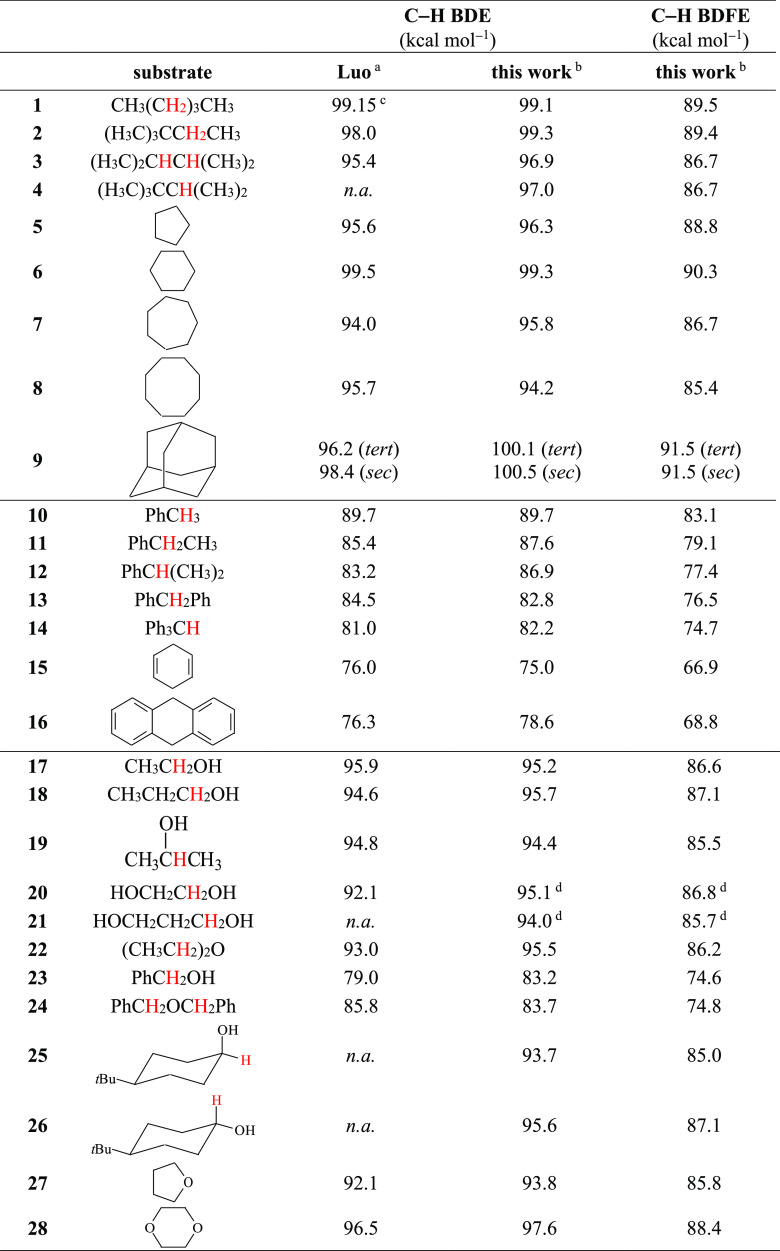

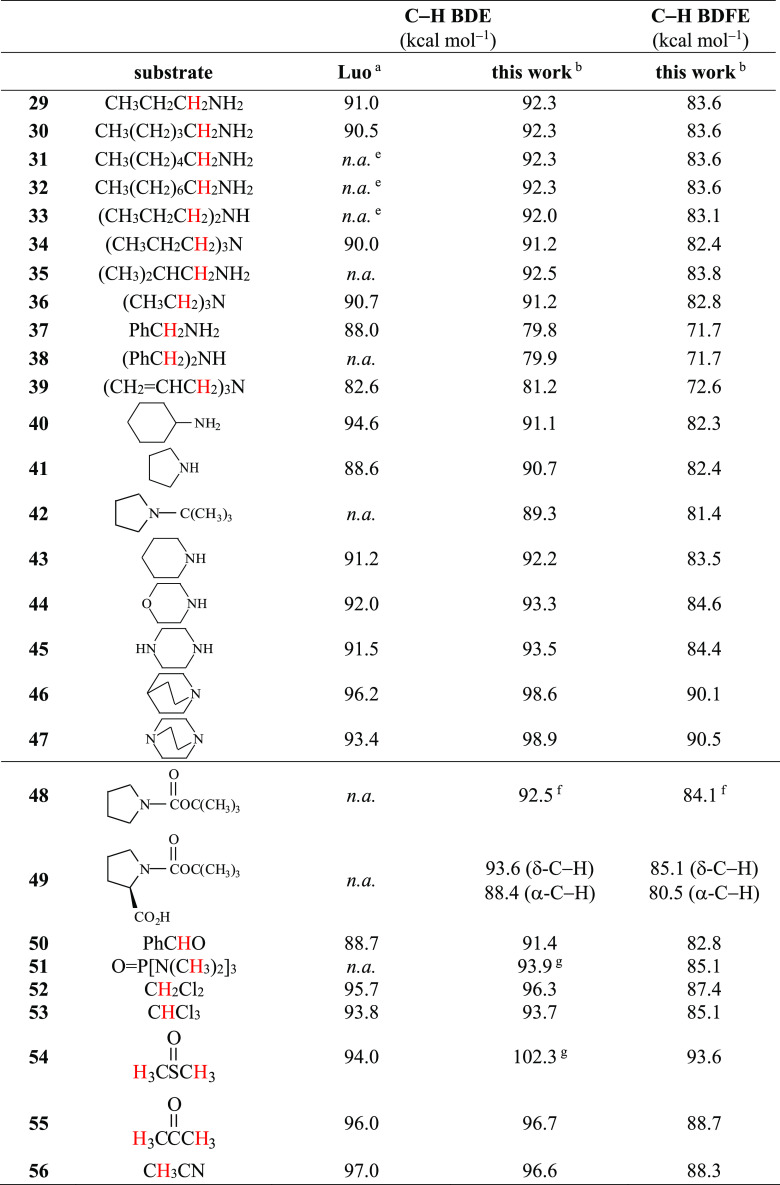
BDE and BDFE Values for the Pertinent C–H Bonds of Substrates **1**–**56**

a Taken from ref (31), recommended values (where available).

b Calculated using the (RO)CBS-QB3 approach (see text).

c Average of the recommended values for the C–H bonds at C-2 and C-3 (99.2 and 99.1 kcal mol^–1^, respectively).

d BDE and BDFE values for the C–H bonds that are α to the HBD OH group in intramolecular hydrogen-bonded structures (see text).

e In Figure 1, BDEs for **31** and **32** were taken to be the same as the value for **30**;^31^ the BDE for **33** was assumed to be the same as the value for **34**.^31^

f Because the calculations lead to slightly different BDEs and BDFEs for the C–H bonds of this substrate that are *cis* and *trans* to the carbonyl group (BDE = 92.6 and 92.3 kcal mol^–1^, BDFE = 84.2 and 83.9 kcal mol^–1^ respectively), the given values are an average of the BDEs and BDFEs for the two C–H bond couples.

g In Figure 1, the BDEs for **51** and **54** were from ref (47) ; see text.

Compared to Luo’s set of compiled data,^31^ the computed BDE for the benzylic C–H bonds of benzylamine (**37**) is significantly lower (79.8 vs 88.0 kcal mol^–1^). The computed value is also similar to that obtained for the corresponding C–H bonds of dibenzylamine (**38**). The computed BDEs for the benzylic C–H bonds of benzyl alcohol (**23**) and dibenzyl ether (**24**) are now very similar, in line with expectations (83.2 and 83.7 kcal mol^–1^, respectively). The computations also produce BDEs for the secondary and tertiary C–H bonds of adamantane (**9**) that are very similar, viz., 100.5 and 100.1 kcal mol^–1^, respectively.

Interestingly, with 1,2-ethanediol (**20**) and 1,3-propanediol (**21**) calculations predict an intramolecular hydrogen-bonded structure that in acetonitrile is more stable than the non-hydrogen-bonded one by 2.5 and 3.5 kcal mol^–1^, respectively (SI, Figure S28). The BDEs for the C–H bonds that are α to the hydrogen bond donor (HBD) and hydrogen bond acceptor (HBA) OH groups are 95.1 and 97.4 kcal mol^–1^ and 94.0 and 96.0 kcal mol^–1^, for **20** and **21**, respectively. The corresponding BDFEs are 86.8 and 88.4 kcal mol^–1^ and 85.7 and 87.2 kcal mol^–1^. Based on these findings, it can be reasonably assumed that with both substrates HAT to CumO^•^ predominantly occurs from the weaker and more electron-rich C–H bonds of a single methylene unit, and accordingly, for these two substrates the *k*_H_′ values displayed in Table 2 have been obtained considering *n* = 2.

Also included in Table 3 are the computed gas-phase C–H BDFEs for substrates **1**–**56**, calculated using the (RO)CBS-QB3 approach. The BDFEs are on average 8.6 ± 0.7 kcal mol^–1^ lower than the corresponding BDEs (uncertainty is 1σ). This difference is primarily due to the entropy of H^•^_(g)_, *T*Δ*S°*(H^•^)_(g)_ = 8.17 kcal mol^–1^.^50^ The agreement between these values indicates that the entropies of R–H and R^•^ are close to the same.^51^ Because there is close to a constant shift between BDE and BDFE, the plots in this report look very similar using either parameter, with just a change in the horizontal axis (see below).

For comparison, the computed O–H BDE and BDFE for cumyl alcohol (2-phenylpropan-2-ol) are 106.6 and 98.2 kcal mol^–1^.

### Rate Constant–Bond Strength Correlations

**Figure 2:**
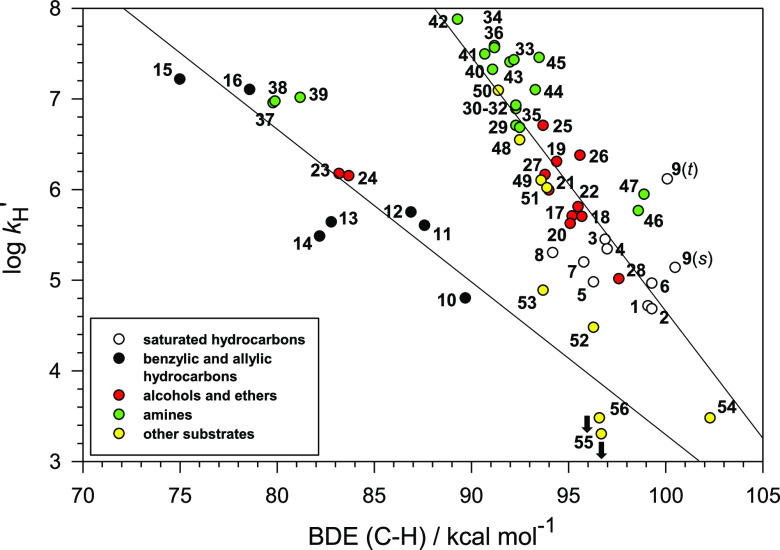
Plot of log *k*_H_^′^ vs C–H BDEs calculated using the (RO)CBS-QB3 approach, for reaction of the cumyloxyl radical (CumO^•^) with substrates **1**–**56**. The log *k*_H_^′^ values for **55** and **56** are upper limits.

In Figure 2, we plot the measured log *k*_H_^′^ values for HAT from substrates **1**–**56** to CumO^•^ taken from Table 1 and Table 2 against the calculated C–H BDEs from Table 3.

Figure 2 reveals trends in the relationship between log *k*_H_^′^ and C–H BDE that are not apparent in Figure 1a, clarifying the trends that are roughly present in Figure 1b. Specifically, the data associated with the benzylic and allylic hydrocarbons (black circles), i.e., the *unsaturated* group, show a relatively good correlation, in particular with the inclusion of the data associated with benzyl alcohol (**23**), dibenzyl ether (**24**), benzylamine (**37**), dibenzylamine (**38**), and triallylamine (**39**). Figure 2 also shows that the points for saturated hydrocarbons, alcohols, ethers, diols, amines, and carbamates (*saturated* group) tend to cluster around a different line with a slope that is steeper than that associated with the benzylic/allylic set, albeit with a lower correlation coefficient. Collectively, Figure 2 demonstrates that, depending on the nature of the substrate, there are two distinct EP relationships, and these results provide strong support for the bimodal behavior observed previously in purely theoretical studies of reactions promoted by other HAT reagents.^16,19^

### Correlation of Δ*G*^⧧^ with Δ*G*°

Recasting the rate/bond strength relationships in terms of bond dissociation *free energies* allows plots such as Figure 2 to be viewed as linear free energy relationships and analyzed with a version of Marcus theory. The basic Marcus equation for the reaction barrier depends only on the free energy driving force (Δ*G*°) and the reorganization energy λ (eq 2). λ is the energy required to reorganize the reactants and their surrounding solvent to the structure of the products without the hydrogen atom transferring. In this model, the barrier at Δ*G*° = 0 is λ/4, which is sometimes termed the “intrinsic barrier” Δ*G*^⧧^_0_. The Brønsted α is then given by eq 3 (assuming λ does not vary with Δ*G*° across the series).
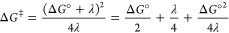
2
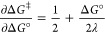
3

**Figure 3:**
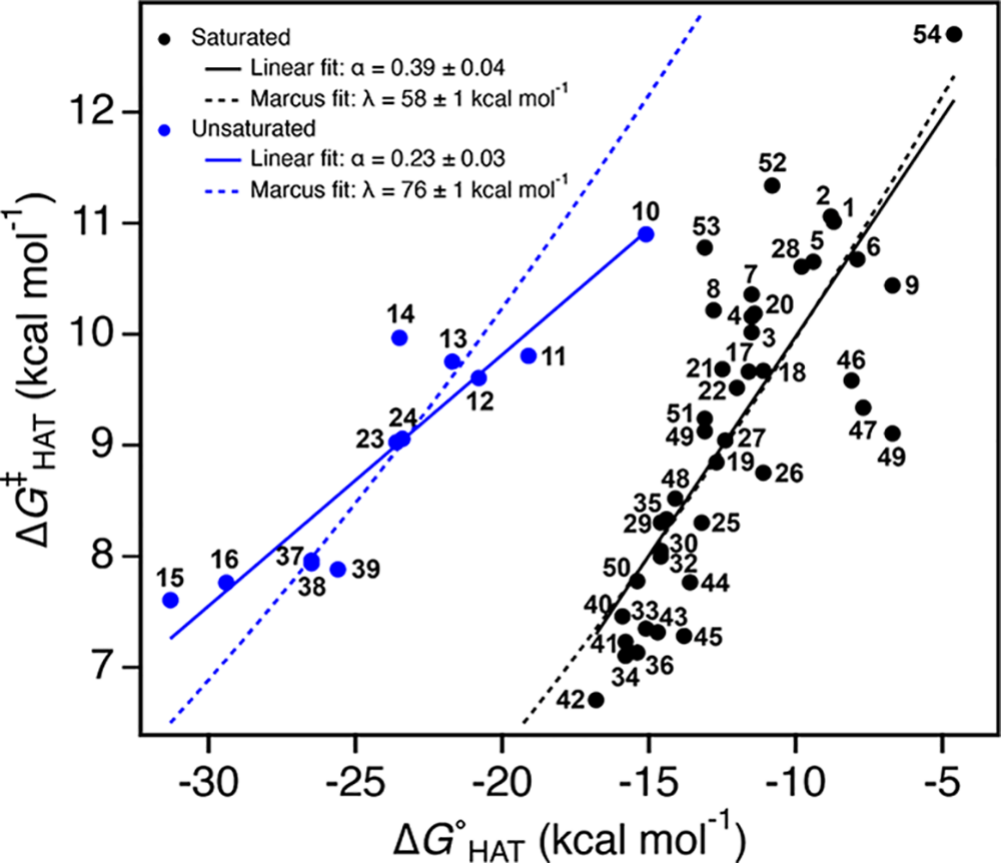
Plot of Δ*G*^⧧^_HAT_ vs Δ*G*°_HAT_ derived from the data in Tables 1–3 (omitting data for **55** and **56**). The black circles are for the *saturated* substrates, and the best linear fit (black solid line) has a Brønsted slope α = 0.39 with an intercept Δ*G*^⧧^_0_ = 13.9 ± 0.6 kcal mol^–1^ at Δ*G*° = 0. The *unsaturated* data are shown in blue, and the fit line has α = 0.23 with Δ*G*^⧧^_0_ = 14.3 ± 0.7 kcal mol^–1^ at Δ*G*° = 0. The best fit to the Marcus equation for the saturated (black dashed curve) and unsaturated (blue dashed curve) substrates is obtained with λ = 58 ± 1 and 76 ± 1 kcal mol^–1^, respectively.

The driving force for the HAT reactions, or Δ*G*°_HAT_, is the computed BDFE for the C–H bond minus BDFE(CumO–H) (since free energies of solution are very similar for RH and R^•^).^52^ The HAT rate constants displayed in Table 1 and Table 2 are converted to Δ*G*^⧧^_HAT_ with the Eyring equation. The plot of Δ*G*^⧧^_HAT_ vs Δ*G*°_HAT_ (Figure 3) is a Brønsted plot, equivalent to a plot of log *k*_H_′ vs log *K*_eq_. Again, *saturated* and *unsaturated* classes of substrates fall on two lines that have different slopes. This is not surprising since very similar data are being plotted (with the vertical axis inverted since log *k*_H_′ ∝ −Δ*G*^⧧^_HAT_).

The slopes of these linear free energy relationships, ∂Δ*G*^⧧^_HAT_/∂Δ*G*°_HAT_ = ∂log *k*_H_′/log *K*_eq_ = Brønsted α, are unitless and carry some intuition. For the unsaturated compounds (solid blue line), α = 0.23, meaning that the barrier is not very sensitive to the driving force: a decrease of 1 kcal mol^–1^ in Δ*G*°_HAT_ only lowers the barrier by 0.23 kcal mol^–1^. The saturated line (solid black line) is steeper, however, with α = 0.39. The intercepts of the two lines, Δ*G*^⧧^_0_ at Δ*G*°_HAT_ = 0, are 14.3 ± 0.7 and 13.9 ± 0.6 kcal mol^–1^ for the unsaturated and saturated substrate groups, respectively. The saturated data show a good fit (dashed black curve) to the Marcus equation (eq 2), with λ = 58 ± 1 kcal mol^–1^. In contrast, the best fit for the unsaturated substrates does not match the slope of the data (blue dashed curve, λ = 76 ± 1 kcal mol^–1^).

## Discussion

### *k*_H_′ vs BDE Correlations

To our knowledge, this is the most extensive experimental data set that has been assembled to examine rate–bond strength correlations for hydrogen atom transfer. A consistent set of BDEs has been assembled using high-level computations. While there is some scatter and some substrates are outliers, the data clearly sort into two primary categories: *saturated* C–H bonds vs *unsaturated* (allylic or benzylic) C–H bonds. This separation is clearly seen both in the log *k*_H_′ vs BDE plot and in the data recast as Δ*G*^⧧^_HAT_ vs Δ*G*°_HAT_ (Figures 2 and 3).

### Outliers from the Correlations

As mentioned above, for acetone (**55**) and acetonitrile (**56**) only an upper limit to *k*_H_ (<1 × 10^4^ M^–1^ s^–1^) could be determined, and accordingly these two substrates were excluded from the correlations. The C–H bonds of acetone and acetonitrile (BDE = 96.7 and 96.6 kcal mol^–1^, respectively) are similar in strength to the tertiary C–H bonds of 2,3-dimethylbutane (**3**) and 2,2,3-trimethylbutane (**4**) (BDE = 96.9 and 97.0 kcal mol^–1^), but the HAT rate constants for **55** and **56** are at least 70 times lower. The extremely low reactivity of these compounds is typically ascribed to an electronic “polar effect” determined by the electron-withdrawing character of the carbonyl and cyano groups.^48^ Such deactivation toward HAT to the electrophilic CumO^•^ prevents the study of substrates bearing C(sp^3^)–H bonds α to strong electron-withdrawing functional groups. Still, it is interesting to note that in Figure 2 the corresponding data points fall significantly closer to the benzylic/allylic correlation line than to the *saturated* one, in agreement with the results obtained in the above-mentioned theoretical studies.^16,19^ Thus, it is possible that the observed deactivation also has a contribution from the factors that make benzylic and allylic C–H bonds less reactive than saturated compounds with the same BDE (see below).

Significant outliers from the best-fit lines displayed in Figure 2 are observed for both the *saturated* and *unsaturated* groups of substrates. Diphenylmethane (**13**) and triphenylmethane (**14**) are less reactive than expected, perhaps due to steric effects. Removing these two substrates from the fit improves the correlation coefficient from 0.755 to 0.905. The most significant outliers from the *saturated* group are adamantane (**9**), 1-azabicyclo[2.2.2]octane (**46**), 1,4-diazabicyclo[2.2.2]octane (**47**), dichloromethane (**52**), and chloroform (**53**). Removing these substrates from the fit improves the correlation coefficient from 0.689 to 0.878.

The measured *k*_H_′ values for HAT from the α-C–H bonds of 1-azabicyclo[2.2.2]octane (**46**) and 1,4-diazabicyclo[2.2.2]octane (**47**) (*k*_H_′ = 5.8 × 10^5^ and 8.8 × 10^5^ M^–1^ s^–1^, respectively) are significantly lower than those associated with conformationally nonrestricted acyclic and cyclic tertiary amines (for which *k*_H_′ = (3–4) × 10^7^ M^–1^ s^–1^).^35,37^ This decrease in reactivity was previously explained on the basis of the operation of stereoelectronic effects.^15,35,49,53^ In **46** and **47**, the α-C–H bonds undergoing HAT and the radical formed are held in conformations that prevent optimal overlap with the nitrogen lone pair, minimizing hyperconjugative α-C–H bond weakening and radical stabilization. The computed BDEs (98.6 and 98.8 kcal mol^–1^, for **46** and **47**, respectively) are about 8 kcal mol^–1^ higher than those obtained for the corresponding bonds of acyclic tertiary amines, suggesting that the operation of stereoelectronic effects is fully reflected therein. Given these much higher C–H BDEs, the *k*_H_′ values for **46** and **47** are actually higher than expected (positive deviations from the correlation line). The unusually electron-rich character of the nitrogen lone pair, which can favor interaction with the electrophilic CumO^•^, and the rigidity of the bicyclic scaffold of these two substrates (see below) may account for the observed behavior.

The results for adamantane (**9**) require a dedicated discussion. The secondary C–H BDE for an acyclic alkane substrate and the *k*_H_′ for HAT from these bonds can be obtained from the average of the values for pentane (**1**) and 2,2-dimethylbutane (**2**) as 99.2 kcal mol^–1^ and 5.0 × 10^4^ M^–1^ s^–1^, respectively (Table 3 and Table 1). Similarly, the tertiary C–H BDE and *k*_H_′ for HAT from this bond can be obtained from the average of the values for 2,3-dimethylbutane (**3**) and 2,2,3-trimethylbutane (**4**) as 97.0 kcal mol^–1^ and 2.5 × 10^5^ M^–1^ s^–1^, neglecting, to a first approximation, the different accessibility of the secondary C–H bonds of **1** and **2** and of the tertiary C–H bonds of **3** and **4** determined by steric effects. On the basis of these averaged values, the secondary and tertiary C–H bonds of **9** appear to be 2.7 and 5.2 times more reactive than their acyclic counterparts, despite the fact that their C–H BDEs are higher by 0.9 and 3.5 kcal mol^–1^, respectively. This is evident in the positive deviation of this substrate (in particular with respect to HAT from the tertiary C–H bonds) from the *saturated* correlation line (Figure 2). This effect can reasonably be explained on the basis of the unhindered nature of the tertiary C–H bonds and the rigidity of the adamantane scaffold, which should reduce the reorganization energy penalty to form the radical. This deviation likely explains why, to the best of our knowledge, **9** has been never included as a hydrogen atom donor substrate in log *k*_H_′ (or Δ*H*^⧧^) vs BDE correlations, even though it is customarily employed as a mechanistic probe in C–H bond oxidation studies.^54,55^

Removing the aforementioned outliers (namely, **13** and **14** for the *unsaturated*, and **9**, **46**, **47**, **52**, and **53** for the *saturated* substrates) from the Δ*G*^⧧^_HAT_ vs Δ*G*°_HAT_ plot displayed in Figure 3 widens the gap between the Brønsted α values for the two correlations, from 0.23 to 0.22 and 0.39 to 0.51 for the unsaturated and saturated substrates, respectively.

### Polar Effects

The *saturated* substrate group shows a good correlation over a broad range of C(sp^3^)–H bonds, spacing from nonactivated to increasingly activated ones such as those α to electron-releasing hydroxyl, alkoxyl, and amino groups. With the exception of dichloromethane (**52**) and chloroform (**53**), which display strongly electronically deactivated C–H bonds, the presence of the heteroatom causes only barely visible systematic deviations from the correlation line. For the electron-rich substrates in our data set, the amines, alcohols, and ethers, polar effects seem to enhance the rate constants. The effect is not large enough to confidently see distinct correlation lines for different classes of saturated compounds, especially given the scatter for the different compounds within a class. However, Figure 2 shows that all but two of the amines lie on or above the correlation line, while all of the saturated hydrocarbons lie on or below the correlation line (except for adamantane (**9**), see above). The polar effect is most evident when comparing compounds displaying reactive C–H bonds with similar BDEs, in other words traversing up a vertical line in Figure 2. For essentially every BDE where comparisons can be made, the order of log *k*_H_^′^ values is hydrocarbons < oxygenates < amines, with very few exceptions. One group of such compounds, with BDE = 93.7 ± 0.5, is listed in Table 4.

**Table 4:**
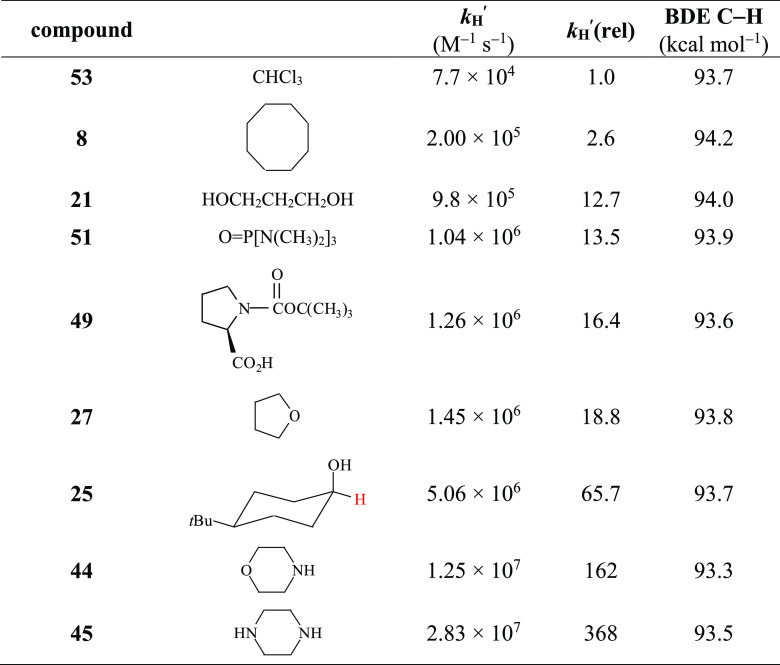
Comparison between the Normalized Rate Constants, *k*_H_^′^, for Compounds with C–H BDEs of 93.7 ± 0.5 kcal mol^–1^

Within this series, chloroform (**53**) displays the lowest *k*_H_^′^ value, in line with the strong C–H bond deactivation determined by the presence of the three electron-withdrawing chlorine atoms. The 142-fold difference between cyclooctane (**8**) and piperazine (**45**) is much larger than the factor of ca. 5 expected for the 1 kcal mol^–1^ difference in BDE between the C–H bonds of these saturated substrates. Thus, the amines react faster than expected even after considering the C–H bond weakening due to hyperconjugation with the nitrogen lone pair. This enhanced reactivity resulting from the neighboring N or O contrasts with the lower reactivity of significantly weaker benzylic and allylic C–H bonds where the product radical can be delocalized over neighboring vinyl or aryl groups. If spin delocalization onto N in the amine substrates were similar to its delocalization in the unsaturated substrates, then the green points in Figure 2 would likely fall between the two correlation lines, not on or above the line for saturated substrates.

*N*-Boc-proline (**49**) provides a striking example of the important role played by polar effects in these reactions.^38^ HAT to CumO^•^ selectively occurs from the δ-C–H bonds next to the N center,^56^ despite the fact that computations indicate that these bonds are stronger than the tertiary α-C–H one by 5.2 kcal mol^–1^. Polar effects resulting from the presence of the CO_2_H group account for α-C–H bond deactivation, while the carbamate nitrogen atom is still sufficiently electron rich to activate the δ-C–H bonds toward HAT.

The *unsaturated* compounds similarly fall close to a correlation line, which covers a similar range of *k*_H_′ values at significantly weaker C–H bonds and has a shallower slope. The amines, alcohol, and ether that are allylic or benzylic fall on this unsaturated line, rather than with the saturated amines or oxygenates.

**Scheme 1:**
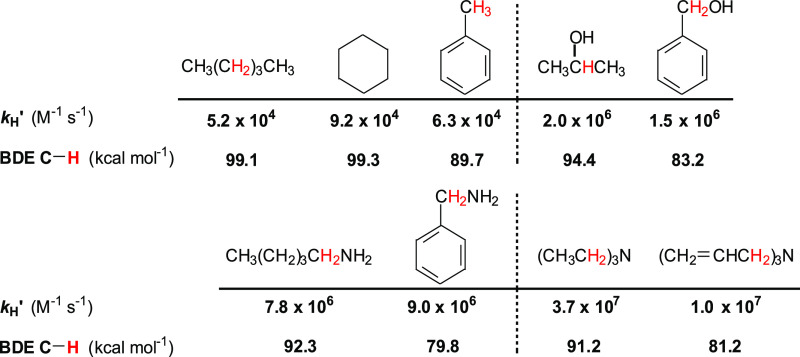
Comparison between C–H BDEs of Different Substrate Groups with Similar Normalized HAT Rate Constants, *k*_H_′

The observed behavior is nicely exemplified by comparing reactions from the two groups with similar *k*_H_′ values but significantly different C–H BDEs (Scheme 1). In all the examples shown, for hydrocarbons, alcohols, and amines, the 9–13 kcal mol^–1^ lower BDEs for the benzylic or allylic C–H bonds remarkably do not lead to higher rate constants than their saturated counterparts. A similar pattern has been observed in HAT reactions promoted by other oxygen-centered abstractors such as the *tert*-butylperoxyl radical (^*t*^BuOO^•^) and photoexcited decatungstate,^28b,57^ as well as in the experimental study of HAT to *t*BuO^•^ by Tanko^15^ and the computational one of HAT to DMDO by Houk,^16^ supporting the generality of these conclusions.

### The Principle of Nonperfect Synchronization as the Origin of the Higher Intrinsic Barriers for Unsaturated Substrates

The sorting of the experimental rate constants for C–H abstraction by CumO^•^ into *saturated* vs *unsaturated* substrates is a deviation from the simple Evans–Polanyi correlation discussed above. This sorting is similar to that made in the theoretical study by Houk and co-workers of C(sp^3^)–H bond oxidations promoted by DMDO.^16^ A similar pattern was seen in early theoretical calculations on HAT reactions from C(sp^3^)–H bonds to the *p*-nitrosophenoxyl radical, where higher intrinsic barriers were suggested for conjugated substrates as compared to the unconjugated counterparts.^19^ In the computational DMDO study, the bimodal EP relationships separated the oxidations of C–H bonds that were aliphatic or α, β, γ, and δ to an oxygen atom (saturated) from those that were benzylic, allylic, or α to C=O or C≡N (unsaturated). Houk et al. explained this separation on the basis of Bernasconi’s principle of nonperfect synchronization,^17^ by suggesting that with the unsaturated substrates resonance stabilization of the product radical is proportionally greater than that for the corresponding transition state.

An analogous PNS explanation can be put forward in the present study, in order to account for the bimodal EP relationship observed for HAT from the C(sp^3^)–H bonds of substrates **1**–**56** to CumO^•^ (Figure 2), grouped analogously. The product stabilizing factor that lowers the BDE—resonance stabilization—develops late along the reaction coordinate, mostly after the transition state. Because the transition state is not proportionally as stabilized as would have been expected on the basis of the BDE, the rates for the resonance-stabilized compounds are slower than expected. The shallower slope observed for the *unsaturated* substrate group is indicative of reactions characterized by an even more imbalanced transition state, where the relative importance of benzylic or allylic resonance stabilization increases on going from the HAT transition state to the product radical (see below). The results reported here and in the Houk theoretical study, together with prior work, suggest that the division in terms of *saturated* and *unsaturated* substrates could represent a general feature in the reactions of oxygen-based HAT reagents with C(sp^3^)–H donors.

### Slopes of the Correlation Lines

The slopes of the correlation lines displayed in Figure 3 provide insights beyond the sorting of *saturated* vs *unsaturated* substrates. The *unsaturated* group falls on a distinctly shallower line, with a unitless slope α = 0.23 for ∂Δ*G*^⧧^/∂Δ*G*°. A shallow slope is also observed in Houk’s computational data set, with α = 0.35 for ∂Δ*H*^⧧^/∂Δ*H*°. The slopes for the *saturated* compounds are both larger, but quite different for the two studies: α_saturated_ = 0.39 for CumO^•^ vs 0.91 for DMDO. These differences in slope could reflect differences in the ranges of driving forces, for instance the large α value for HAT from *saturated* substrates to DMDO, reflecting that all of the reactions considered in that group were endothermic. The more exergonic reactions should have earlier transition states and therefore shallower slopes, according to the Hammond postulate. However, this explanation should have resulted in curved correlations, given the wide range of driving forces in the studies, but this was not observed. More insight from the slopes can be derived from the Marcus-type analysis in the next section.

### Marcus Theory and Valence Bond Analyses of Reorganization Energies

A model based on Marcus theory has been shown by one of us to reasonably well predict many HAT rate constants, within an order of magnitude or two.^3b,58^ In the Δ*G*^⧧^_HAT_ vs Δ*G*°_HAT_ plot displayed in Figure 3, the line for the *saturated* substrates is very well described by this model. The reorganization energy λ can be estimated in two different ways. The best fit of the set of *saturated* points (Δ*G*^⧧^_HAT_, Δ*G*°_HAT_) to eq 2 (black dashed curve in Figure 3) gives λ = 58 ± 1 kcal mol^–1^. Alternatively, extrapolating the linear correlation to Δ*G*°_HAT_ = 0 gives Δ*G*^⧧^_0_ = 13.9 ± 0.6 kcal mol^–1^, which is λ/4, giving λ = 56 ± 2 kcal mol^–1^. The agreement between these values and with the data points shows that the simple Marcus model fits these data very well. We emphasize that this good fit is a significant result, not just a fitting exercise: the correlation line has two variables (slope and intercept), while the Marcus equation (eq 2) has a specific functional form and only a single parameter (λ). The single λ sets both the width and intercept of the Marcus parabola for Δ*G*^⧧^_HAT_. The success of the simple Marcus model is remarkable.

However, this Marcus theory model with a constant λ *does not* describe the data for the unsaturated substrates. The best fit of eq 2 to the unsaturated data (blue dashed curve in Figure 3) is much too steep. The fit has λ = 76 ± 1 kcal mol^–1^, dramatically higher than the extrapolation from the linear fit to get Δ*G*^⧧^_0_(unsaturated) = 14.3 ± 0.7 kcal mol^–1^, which would imply λ = 57 ± 3 kcal mol^–1^ (see the SI). Despite the unsaturated data being on a very different line, the Δ*G*°_HAT_ = 0 *intercept* for their correlation line is within error of the saturated intercept. While the Marcus fit is poor, it is clear that the unsaturated compounds must have larger λ’s since they have similar rate constants at larger driving forces. Fitting just the fastest or slowest unsaturated points to the Marcus equation gives λ values of ∼68 or ∼78 kcal mol^–1^, respectively. Thus, if these data are to be fit within the Marcus picture, *the reorganization energy must vary with driving force* for the unsaturated compounds. Specifically, the λ’s for unsaturated compounds must be *greater* than the λ’s for saturated ones, and the unsaturated λ’s must increase as the C–H bonds become weaker. In this model, the increase in λ offsets the increase in driving force, leading to the shallower slope for the unsaturated substrates. This pattern of changes in λ is confirmed by computations of the Marcus inner-sphere reorganization energies λ_i_ using a version of Nelsen’s four-point approach (see the SI for details).^59,60^ The computed λ_i_ increased from saturated pentane (24.5 kcal mol^–1^) to unsaturated toluene (39.1 kcal mol^–1^) and cyclohexadiene (52.4 kcal mol^–1^). The same trend was seen with the increase in λ_i_ from 2-propanol (19.7 kcal mol^–1^) to benzyl alcohol (58.5 kcal mol^–1^).

Unsaturated substrates having higher barriers than saturated ones was predicted by Shaik and co-workers, using semiempirical valence bond state correlation diagrams.^20,21^ This model uses vertical bond strengths *D*_H–Y_ and reorganization energies for relaxation of the radicals (Y^•^) to their preferred geometry when free (−RE_Y•_). The thermodynamic BDE is then *D*_H–Y_ – RE_Y•_. Using the semiempirical VBSCD approach, they show that the “intrinsic barrier” for a HAT reaction, the barrier at zero driving force Δ*E*^⧧^_VB,0_, has a significant contribution from the RE terms (eq 4).^21c^ The VB reorganization energy and intrinsic barrier are not the same as those parameters in Marcus theory, but they are related. The RE terms for alkanes were computed to be ∼7 kcal mol^–1^, while those for propene, toluene, and ethylbenzene were larger (16.7, 12.3, and 18.4 kcal mol^–1^, respectively).^21c^ While not predictive in detail (ethylbenzene is more reactive than toluene), this equation captures the distinction between saturated and unsaturated substrates.
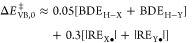
4

The variations in λ and the VB RE provide a qualitative connection between the PNS and Marcus models. Bernasconi himself noted that “a recurrent theme will be that high intrinsic barriers are typically associated with a lack of synchronization between concurrent reaction events such as bond formation/cleavage, solvation/desolvation, development (loss) of resonance, etc*.*”^17c^ The higher reorganization energies for HAT from benzylic/allylic C–H bonds as compared to aliphatic C–H bonds likely reflect the delocalization of the product radical in the unsaturated group, requiring bond length changes in a number of relatively high-frequency modes. Within the unsaturated group, the compounds with the weaker C–H bonds have more radical stabilization typically due to more extensive delocalization. Therefore, the reorganization energy should become larger as the Δ*G*°_HAT_ becomes more favorable, as observed. In this picture, the PNS occurs because of the balance between energetic cost in reorganization and the favorable radical stabilization.

The Marcus and VB reorganization energies provide some intuition about the origins of some of the outliers from the correlations in Figures 2 and 3. Within the set of *saturated* substrates, for example, the largest outliers on the faster side are the most rigid ones: adamantane (**9**) and 1-aza- and 1,4-diazabicyclo[2.2.2]octanes (**46** and **47**). Presumably the rigidity of these substrates limits the reorganization that can occur. The point for benzaldehyde (**50**), on the other hand, falls on the saturated correlation line, a behavior that reasonably reflects the localized nature of the σ-radical formed following abstraction of the formylic hydrogen.^39^

It is interesting to compare radical stabilization by a vinyl or aryl group with that from a heteroatom lone pair as in an amine. If it were just the presence of radical stabilization that increased λ, then the amine points would be expected to have rate constants below the saturated correlation line, but the opposite is observed: hyperconjugation places the rate constants above the correlation line. The acceleration caused by the nitrogen heteroatom does not appear to be subject to the PNS, indicating a distinction between the stabilization provided by a π-system and a heteroatom lone pair. Perhaps radical stabilization by a nitrogen lone pair does not involve substantial reorganization because the radical delocalization is quite limited, in contrast to the delocalization of the product radical in the benzylic/allylic substrates, which involve a larger number of significant bond length changes.

## Conclusions

An extensive experimental data set of normalized second-order rate constants *k*_H_′ for HAT from the C–H bonds of 56 substrates to CumO^•^, spanning a range of more than 4 orders of magnitude in reactivity, has been assembled to analyze rate–bond strength correlations. Because of large discrepancies in some of the available C–H BDEs, and the absence of BDEs for some of these substrates, a corresponding set of consistent gas-phase C–H BDEs and BDFEs spanning a range of 27 kcal mol^–1^ has been calculated. Analysis in terms of Evans–Polanyi log *k*_H_′ vs BDE and Marcus-type Δ*G*^⧧^_HAT_ vs Δ*G*°_HAT_ plots shows in both cases the existence of two distinct correlations, one for substrates bearing benzylic and allylic C–H bonds (*unsaturated* group) and the other one for saturated hydrocarbons, alcohols, ethers, diols, amines, and carbamates (*saturated* group). Such bimodal behavior supports previous results from theoretical studies of reactions promoted by other HAT reagents and has been rationalized in terms of Bernasconi’s principle of nonperfect synchronization and Marcus theory. In the unsaturated substrate group, resonance stabilization of the product radical is proportionally greater than that for the corresponding transition state and, as compared to saturated substrates, higher HAT reorganization energies are required. As a result, the significant increase in C–H BDE observed on going from benzylic and allylic to aliphatic hydrogen atom donor substrates does not translate into higher rate constants for the former group. By establishing a qualitative connection between the PNS and Marcus models, the results presented in this study expand previous findings, providing a general framework for a detailed description of the factors that govern HAT reactions from C(sp^3^)–H bonds, possibly representing a stimulus for a deeper understanding and for future development of this important class of reactions.

## Experimental Section

### Materials

Spectroscopic grade acetonitrile and isooctane were used in the kinetic experiments. 2,2-Dimethylbutane (**2**), 2,3-dimethylbutane (**3**), 2,2,3-trimethylbutane (**4**), cycloheptane (**7**), adamantane (**9**), toluene (**10**), ethylbenzene (**11**), cumene (**12**), diphenylmethane (**13**), triphenylmethane (**14**), 9,10-dihydroanthracene (**16**), diethyl ether (**22**), benzyl alcohol (**23**), 1,4-dioxane (**28**), 1-azabicyclo[2.2.2]octane (**46**), 1,4-diazabicyclo[2.2.2]octane (**47**), and benzaldehyde (**50**) were of the highest commercial quality available and were used as received. Commercial samples of propylamine (**29**), hexylamine (**31**), octylamine (**32**), triallylamine (**39**), dichloromethane (**52**), and chloroform (**53**) were purified prior to use by filtration over neutral alumina. The purity of the substrates was checked by GC prior to the kinetic experiments and was in all cases >99.5%. Dicumyl peroxide was of the highest commercial quality available and was used as received.

### Laser Flash Photolysis Studies

LFP experiments were carried out with a laser kinetic spectrometer using the third harmonic (355 nm) of a Q-switched Nd:YAG laser, delivering 8 ns pulses. The laser energy was adjusted to ≤10 mJ/pulse by the use of the appropriate filter. A 3.5 mL Suprasil quartz cell (10 mm × 10 mm) was used in all experiments. Argon- or nitrogen-saturated acetonitrile or isooctane solutions of dicumyl peroxide (1.0 M) were employed. All the experiments were carried out at *T* = 25 ± 0.5 °C under magnetic stirring. The observed rate constants (*k*_obs_) were obtained by averaging 3–5 individual values and were reproducible to within 5%. Second-order rate constants for the reactions of the cumyloxyl radical with the hydrogen atom donor substrates were obtained from the slopes of the *k*_obs_ (measured following the decay of the cumyloxyl radical visible absorption band at 490 nm) vs [substrate] plots. Correlation coefficients were generally >0.99. The given rate constants are the average of at least two independent experiments, with typical errors being ≤5%.

### Computational Methods

All calculations were performed using the Gaussian 09^61^ and Gaussian 16^62^ suite of programs. Conformational searches were performed using RDKit^63^ and Hyperchem,^64^ on all molecular and radical species for which there was uncertainty as to the minimum energy structure. Calculations were performed in order to determine the bond dissociation enthalpy for the weakest C–H bond using the ROCBS-QB3 method.^65^ In cases where it was feasible, W1BD^66^ was used for benchmarking purposes (see the SI). In all cases, structures were verified to be local minima and possessed positive vibration frequencies. Calculation of λ_i_ values was performed using the B3LYP/6-311G(2d,d,p) method.^67,68^

## Supporting Information Available

Plots of *k*_obs_ vs substrate concentration for the reactions of CumO^•^, details of the Marcus analysis, and results of benchmarking studies on C–H BDEs (PDF)
Thermochemical data and *xyz* files (ZIP)
