# Supplementary material for: Bimodal Evans–Polanyi Relationships in Hydrogen Atom Transfer from C(sp3)–H Bonds to the Cumyloxyl Radical. A Combined Time-Resolved Kinetic and Computational Study
Source: J Am Chem Soc. 2021 Jul 26;143(30):11759–76. doi: 10.1021/jacs.1c05566 (PMC8343544; doi:10.1021/jacs.1c05566)
Supplement: Supplementary file 1 — ja1c05566_si_001.pdf [file ja1c05566_si_001.pdf]

SUPPORTING INFORMATION

Bimodal Evans−Polanyi Relationships in Hydrogen Atom Transfer from C(sp^3)–H Bonds to the Cumyloxyl Radical. A Combined Time-Resolved Kinetic and Computational Study.

Michela Salamone,[a] Marco Galeotti,[a] Eduardo Romero-Montalvo,[b] Jeffrey Van Santen,[b] Benjamin D. Groff,[c] James M. Mayer,*[,c] Gino A. DiLabio,*[,b] and Massimo Bietti*[,a]

[a] *Dipartimento di Scienze e Tecnologie Chimiche, Università "Tor Vergata", Via della Ricerca Scientifica, 1 I-00133 Rome, Italy.*

[b] *Department of Chemistry, The University of British Columbia, 3247 University Way, Kelowna, British Columbia, Canada, V1V 1V7*

[c] *Department of Chemistry, Yale University, 225 Prospect St. New Haven, CT, USA 06520-8107*

1) **log k_H' vs C–H BDE plots for data taken from ref. S1** S2
2) **Time-resolved kinetic studies: k_{obs} vs [substrate] plots** S3
3) **Table of k_H, k_H' and C–H BDE values employed for Figure 1** S14
4) **Product studies on the reaction of CumO• with adamantane** S17
5) **Marcus analysis** S18
6) **Plots of ΔG^0_{HAT} *vs* IP and $\Delta G^{\neq}_{HAT}$ *vs* IP** S19
7) **Theoretical calculations** S20
    7.1) **Benchmarking** S20
    7.2) **Marcus' inner-sphere reorganization energies** S23
    7.3) **Structures and thermochemical data** S24
8) **References** S25

1) log k_H' vs C–H BDE plots for data taken from ref. S1

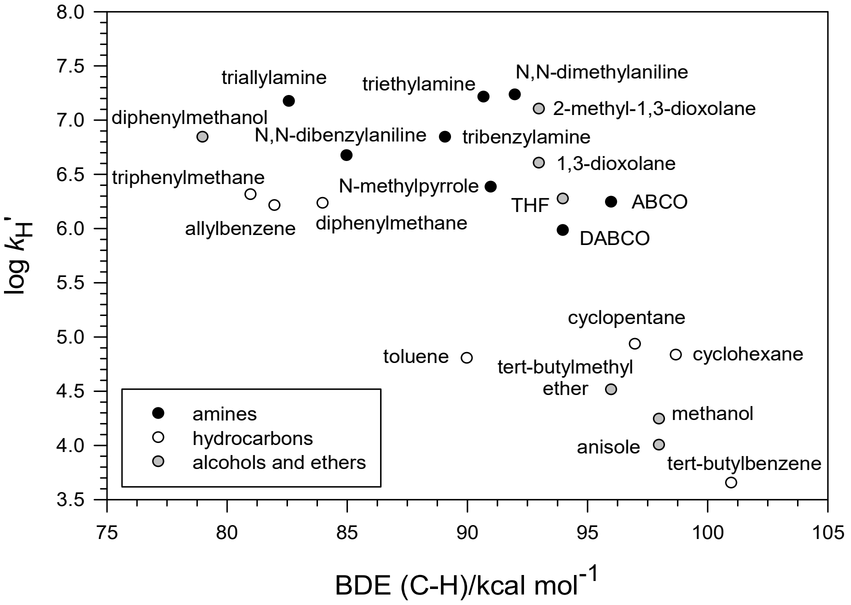

Figure S1. Plot of log k_H' vs C–H BDEs for reaction of the *tert*-butoxyl radical (*t*BuO$^•$) with the indicated substrates, grouped on the basis of amine (black circles), hydrocarbon (white circles), and alcohol and ether (grey circles) C–H bond donor substrates. Data points taken from ref. S1.

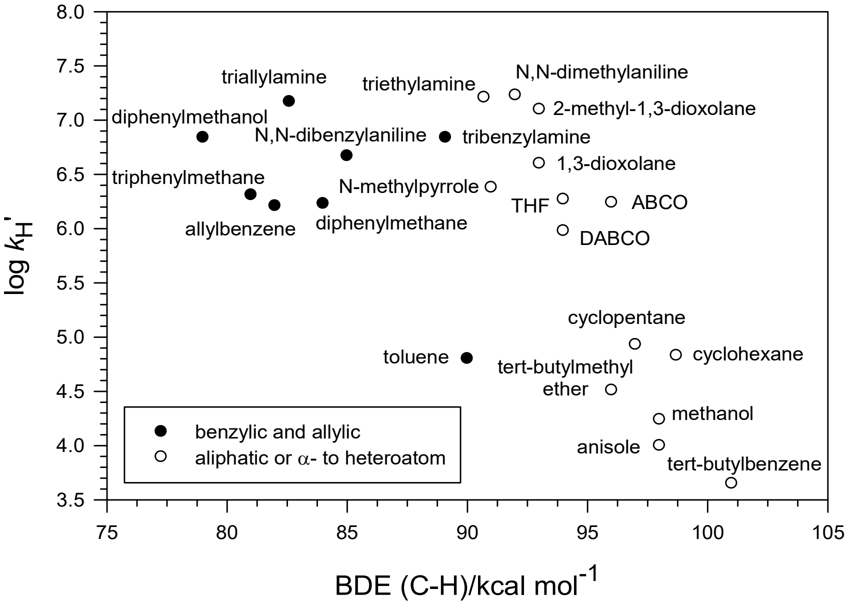

Figure S2. Plot of log k_H' vs C–H BDEs for reaction of the *tert*-butoxyl radical (*t*BuO$^•$) with the indicated substrates. Same data points as in Figure S1, grouped on the basis of substrates bearing benzylic and allylic (black circles) or aliphatic and α- to heteroatom (white circles) C–H bonds.

2) Time-resolved kinetic studies: k_{obs} vs [substrate] plots

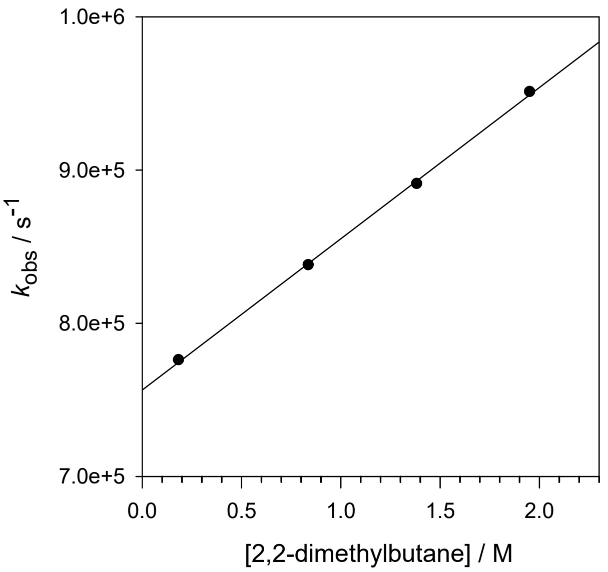

Figure S3. Plot of the observed rate constant (k_{obs}) against [substrate] for the reaction of 2,2-dimethylbutane (**2**) with CumO• generated by 355 nm LFP of an Ar-saturated MeCN solution containing 1.0 M dicumyl peroxide measured at T = 25 °C following the decay of CumO• at 490 nm. From the linear regression analysis: intercept = 7.56×10^5 s^{-1}, $k_H = 9.87 \times 10^4$ M^{-1} s^{-1}, r^2 = 0.9994.

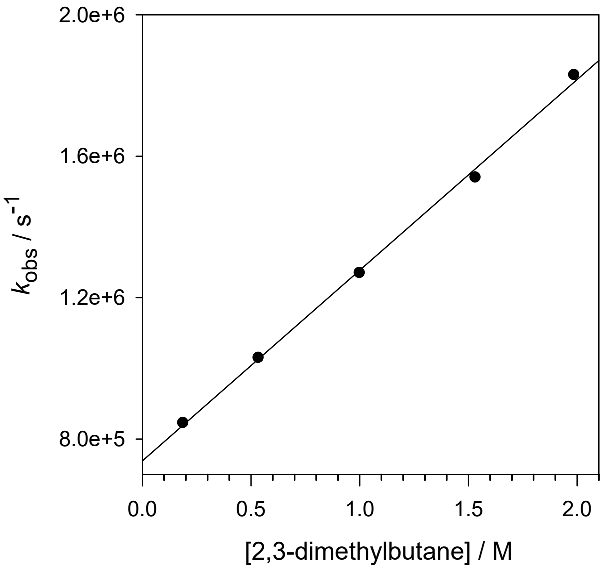

Figure S4. Plot of the observed rate constant (k_{obs}) against [substrate] for the reaction of 2,3-dimethylbutane (**3**) with CumO• generated by 355 nm LFP of an Ar-saturated MeCN solution containing 1.0 M dicumyl peroxide measured at T = 25 °C following the decay of CumO• at 490 nm. From the linear regression analysis: intercept = 7.38×10^5 s^{-1}, $k_H = 5.39 \times 10^5$ M^{-1} s^{-1}, r^2 = 0.9982.

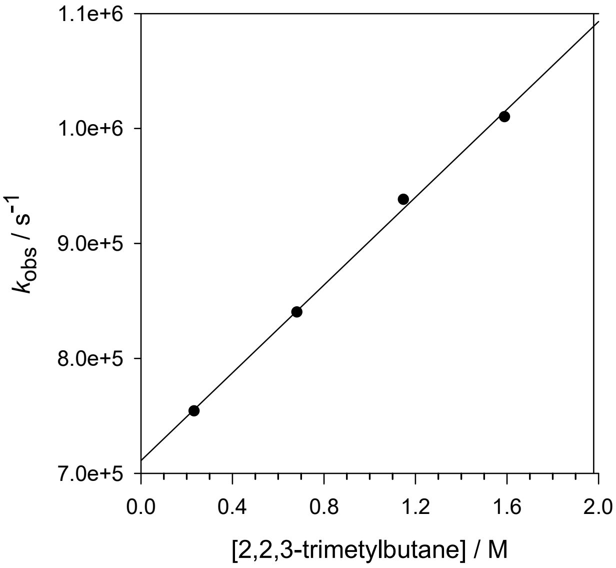

Figure S5. Plot of the observed rate constant (k_{obs}) against [substrate] for the reaction of 2,2,3-trimethylbutane (**4**) with CumO$^{\bullet}$ generated by 355 nm LFP of an Ar-saturated MeCN solution containing 1.0 M dicumyl peroxide measured at T = 25 °C following the decay of CumO$^{\bullet}$ at 490 nm. From the linear regression analysis: intercept = 7.11 × 10^5 s^{-1}, k_H = 1.91 × 10^6 M^{-1} s^{-1}, r^2 = 0.9977.

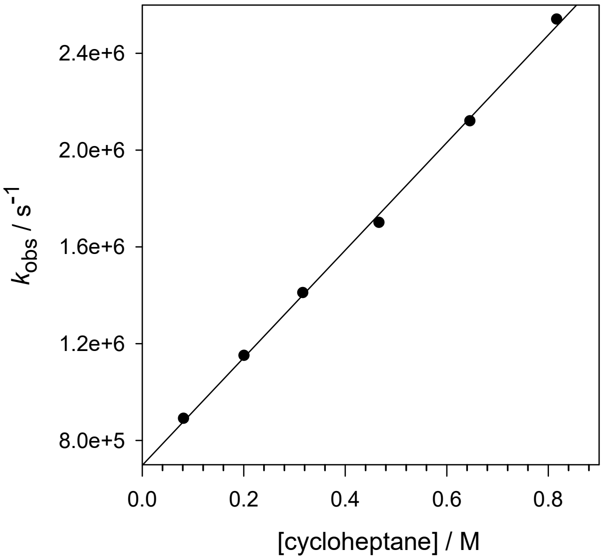

Figure S6. Plot of the observed rate constant (k_{obs}) against [substrate] for the reaction of cycloheptane (**7**) with CumO$^{\bullet}$ generated by 355 nm LFP of an Ar-saturated MeCN solution containing 1.0 M dicumyl peroxide measured at T = 25 °C following the decay of CumO$^{\bullet}$ at 490 nm. From the linear regression analysis: intercept = 6.97 × 10^5 s^{-1}, k_H = 2.22 × 10^6 M^{-1} s^{-1}, r^2 = 0.9988.

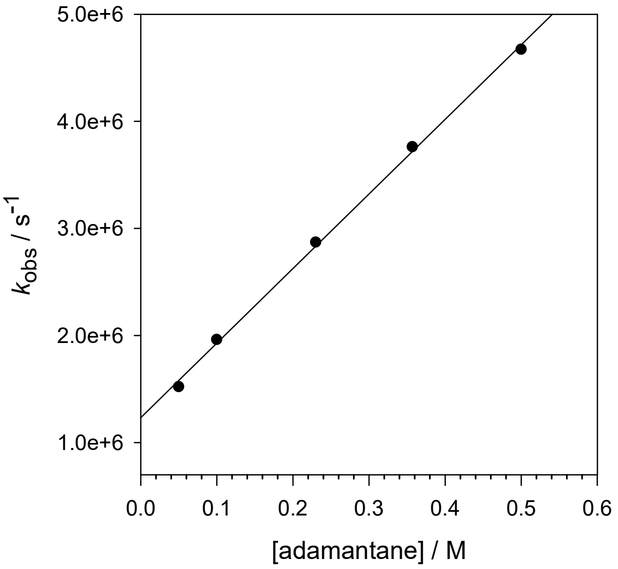

Figure S7. Plot of the observed rate constant (k_{obs}) against [substrate] for the reaction of adamantane (**9**) with CumO• generated by 355 nm LFP of an Ar-saturated isooctane solution containing 1.0 M dicumyl peroxide measured at T = 25 °C following the decay of CumO• at 490 nm. From the linear regression analysis: intercept = 1.23×10^6 s^{-1}, $k_H = 6.96 \times 10^6$ M^{-1} s^{-1}, r^2 = 0.9986.

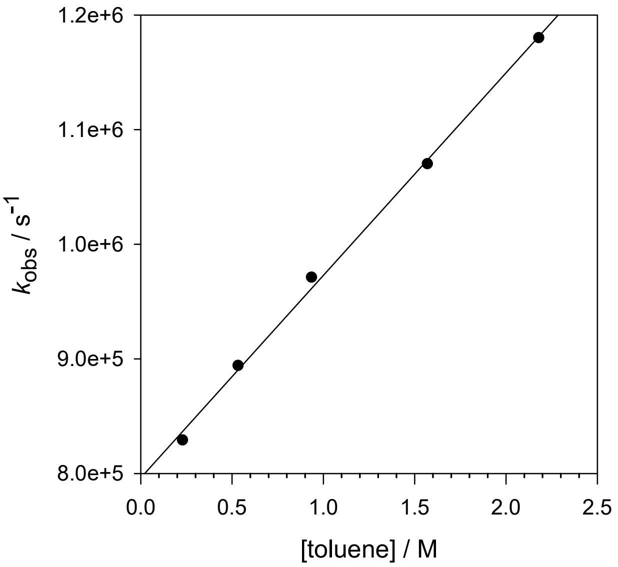

Figure S8. Plot of the observed rate constant (k_{obs}) against [substrate] for the reaction of toluene (**10**) with CumO• generated by 355 nm LFP of an Ar-saturated MeCN solution containing 1.0 M dicumyl peroxide measured at T = 25 °C following the decay of CumO• at 490 nm. From the linear regression analysis: intercept = 7.95×10^5 s^{-1}, $k_H = 1.77 \times 10^5$ M^{-1} s^{-1}, r^2 = 0.9977.

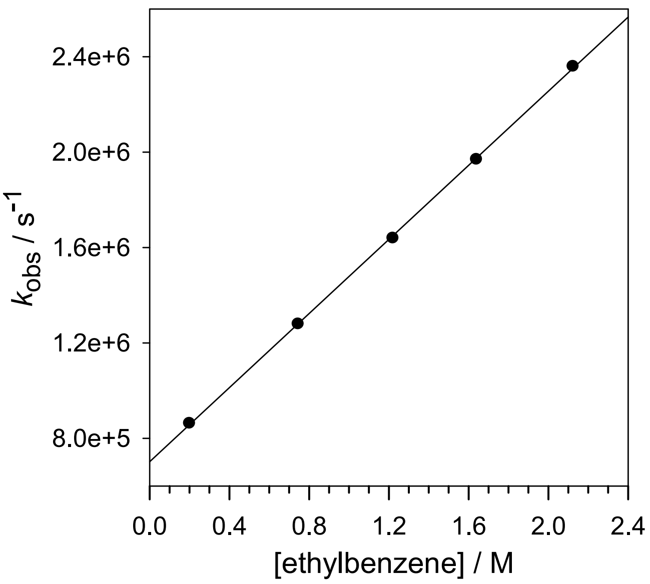

Figure S9. Plot of the observed rate constant (k_{obs}) against [substrate] for the reaction of ethylbenzene (**11**) with CumO$^{\bullet}$ generated by 355 nm LFP of an Ar-saturated MeCN solution containing 1.0 M dicumyl peroxide measured at T = 25 °C following the decay of CumO$^{\bullet}$ at 490 nm. From the linear regression analysis: intercept = 7.02 × 10^5 s^{-1}, k_H = 7.77 × 10^5 M^{-1} s^{-1}, r^2 = 0.9998.

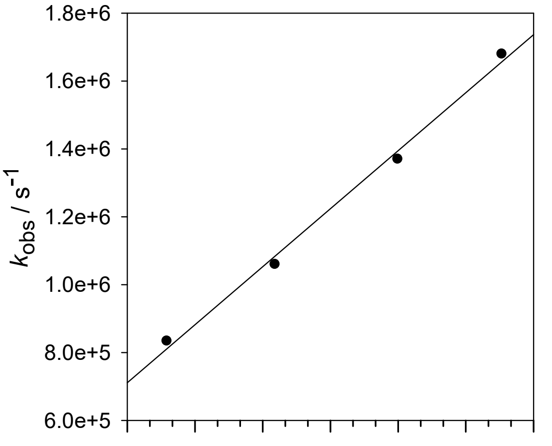

Figure S10. Plot of the observed rate constant (k_{obs}) against [substrate] for the reaction of cumene (**12**) with CumO$^{\bullet}$ generated by 355 nm LFP of an Ar-saturated MeCN solution containing 1.0 M dicumyl peroxide measured at T = 25 °C following the decay of CumO$^{\bullet}$ at 490 nm. From the linear regression analysis: intercept = 7.11 × 10^5 s^{-1}, k_H = 5.70 × 10^5 M^{-1} s^{-1}, r^2 = 0.9946.

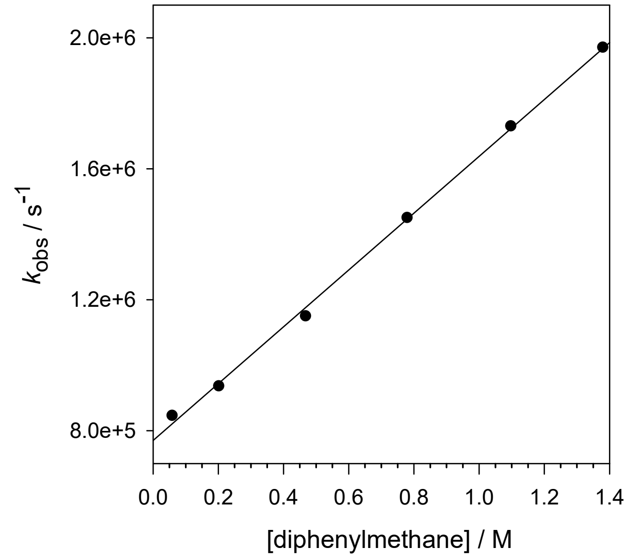

Figure S11. Plot of the observed rate constant (k_{obs}) against [substrate] for the reaction of diphenylmethane (**13**) with CumO$^•$ generated by 355 nm LFP of an Ar-saturated MeCN solution containing 1.0 M dicumyl peroxide measured at T = 25 °C following the decay of CumO$^•$ at 490 nm. From the linear regression analysis: intercept = 7.70 × 10^5 s^{-1}, k_H = 8.68 × 10^5 M^{-1} s^{-1}, r^2 = 0.9985.

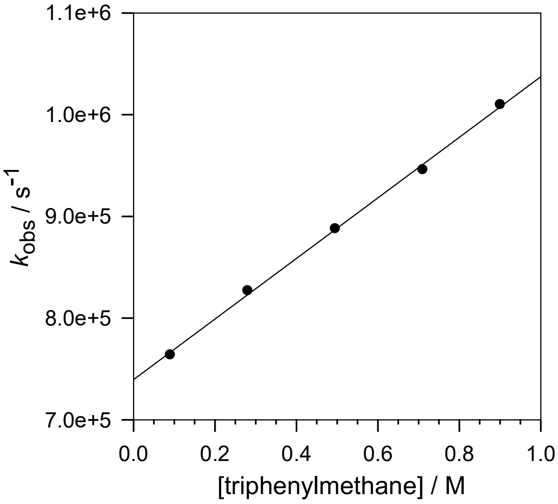

Figure S12. Plot of the observed rate constant (k_{obs}) against [substrate] for the reaction of triphenylmethane (**14**) with CumO$^•$ generated by 355 nm LFP of an Ar-saturated MeCN solution containing 1.0 M dicumyl peroxide measured at T = 25 °C following the decay of CumO$^•$ at 490 nm. From the linear regression analysis: intercept = 7.40 × 10^5 s^{-1}, k_H = 2.98 × 10^5 M^{-1} s^{-1}, r^2 = 0.9986.

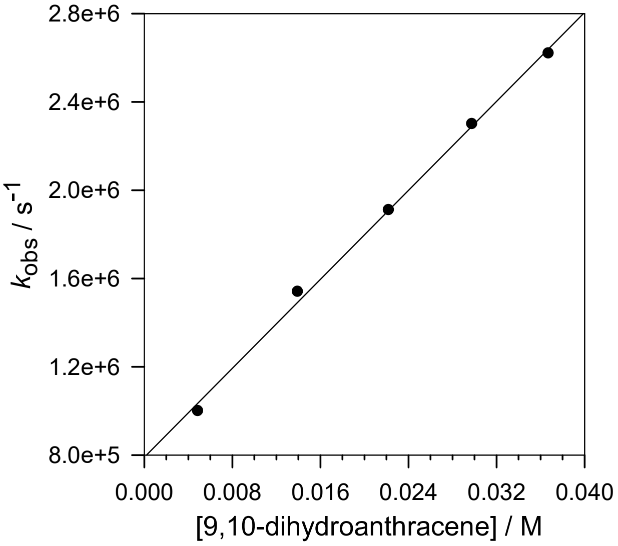

Figure S13. Plot of the observed rate constant (k_{obs}) against [substrate] for the reaction of 9,10-dihydroanthracene (**16**) with CumO$^\bullet$ generated by 355 nm LFP of an Ar-saturated MeCN solution containing 1.0 M dicumyl peroxide measured at T = 25 °C following the decay of CumO$^\bullet$ at 490 nm. From the linear regression analysis: intercept = 7.90 × 10^5 s^{-1}, k_H = 5.04 × 10^7 M^{-1} s^{-1}, r^2 = 0.9975.

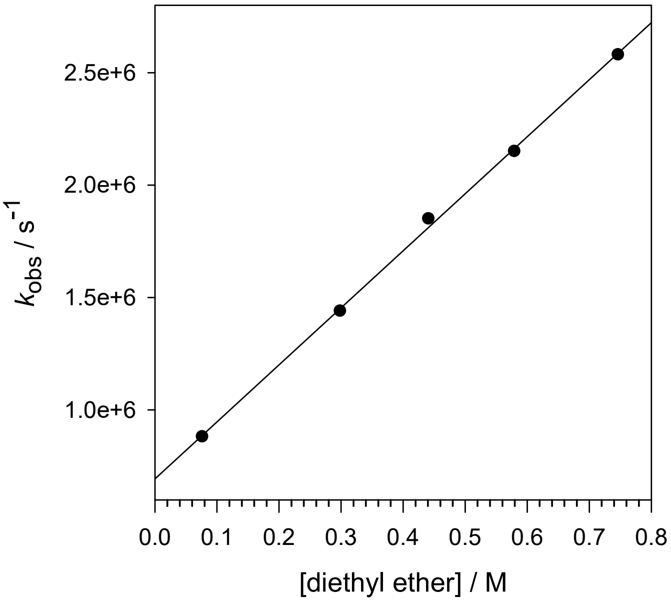

Figure S14. Plot of the observed rate constant (k_{obs}) against [substrate] for the reaction of diethyl ether (**22**) with CumO$^\bullet$ generated by 355 nm LFP of an Ar-saturated MeCN solution containing 1.0 M dicumyl peroxide measured at T = 25 °C following the decay of CumO$^\bullet$ at 490 nm. From the linear regression analysis: intercept = 6.93 × 10^5 s^{-1}, k_H = 2.54 × 10^6 M^{-1} s^{-1}, r^2 = 0.9989.

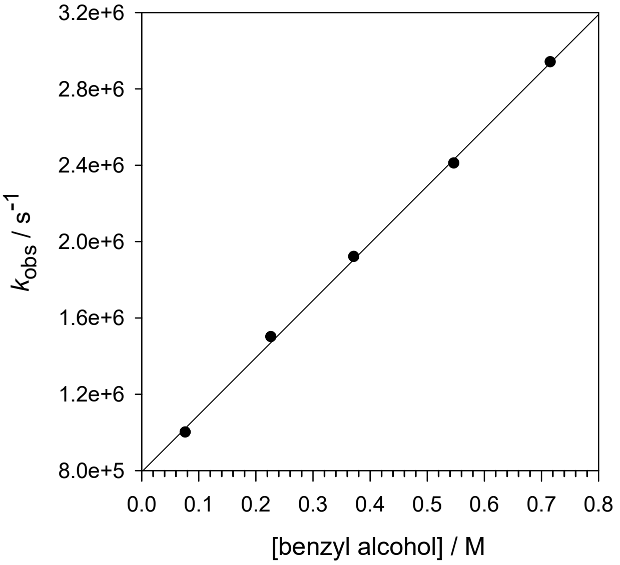

Figure S15. Plot of the observed rate constant (k_{obs}) against [substrate] for the reaction of benzyl alcohol (**23**) with CumO$^{\bullet}$ generated by 355 nm LFP of an Ar-saturated MeCN solution containing 1.0 M dicumyl peroxide measured at T = 25 °C following the decay of CumO$^{\bullet}$ at 490 nm. From the linear regression analysis: intercept = 7.93×10^5 s^{-1}, $k_H = 2.99 \times 10^6$ M^{-1} s^{-1}, $r^2 = 0.9991$.

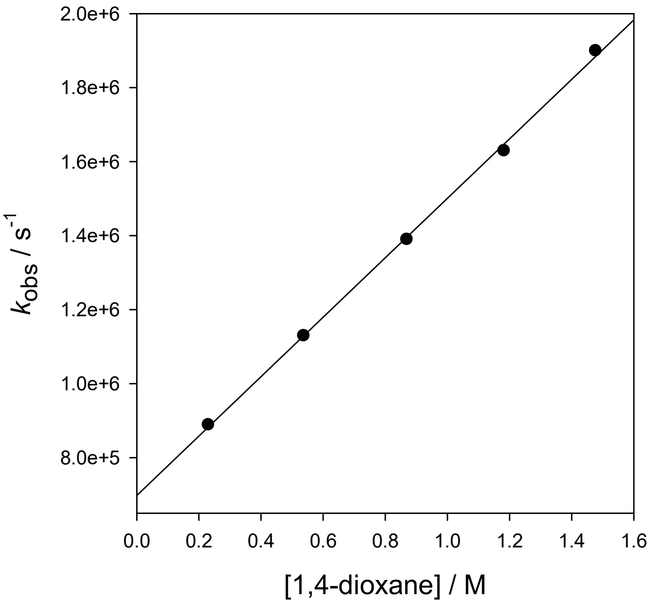

Figure S16. Plot of the observed rate constant (k_{obs}) against [substrate] for the reaction of 1,4-dioxane (**28**) with CumO$^{\bullet}$ generated by 355 nm LFP of an Ar-saturated MeCN solution containing 1.0 M dicumyl peroxide measured at T = 25 °C following the decay of CumO$^{\bullet}$ at 490 nm. From the linear regression analysis: intercept = 6.98×10^5 s^{-1}, $k_H = 8.03 \times 10^5$ M^{-1} s^{-1}, $r^2 = 0.9990$.

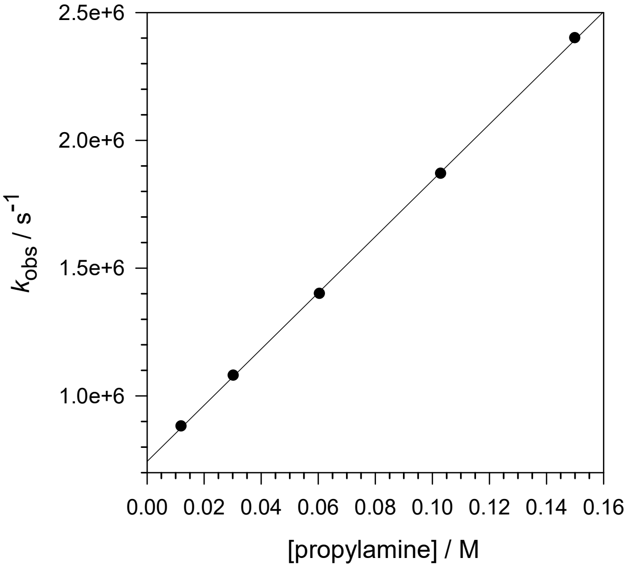

Figure S17. Plot of the observed rate constant (k_{obs}) against [substrate] for the reaction of propylamine (**29**) with CumO$^•$ generated by 355 nm LFP of an Ar-saturated MeCN solution containing 1.0 M dicumyl peroxide measured at T = 25 °C following the decay of CumO$^•$ at 490 nm. From the linear regression analysis: intercept = 7.44×10^5 s^{-1}, $k_H = 1.10 \times 10^7$ M^{-1} s^{-1}, r^2 = 0.9998.

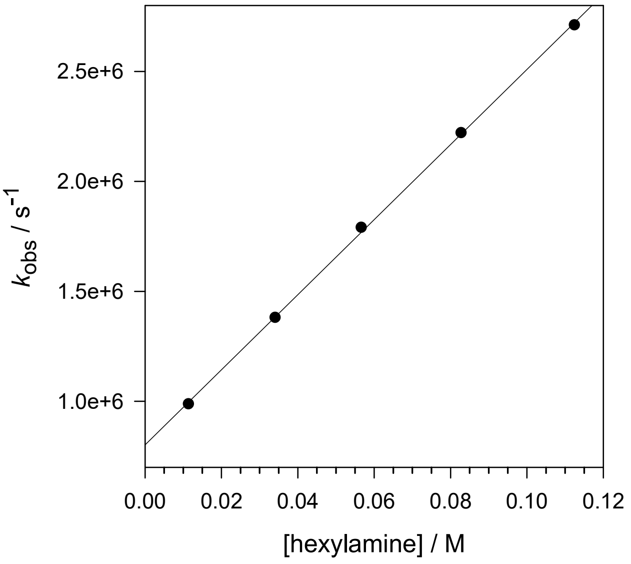

Figure S18. Plot of the observed rate constant (k_{obs}) against [substrate] for the reaction of hexylamine (**31**) with CumO$^•$ generated by 355 nm LFP of an Ar-saturated MeCN solution containing 1.0 M dicumyl peroxide measured at T = 25 °C following the decay of CumO$^•$ at 490 nm. From the linear regression analysis: intercept = 8.02×10^5 s^{-1}, $k_H = 1.71 \times 10^7$ M^{-1} s^{-1}, r^2 = 0.9996.

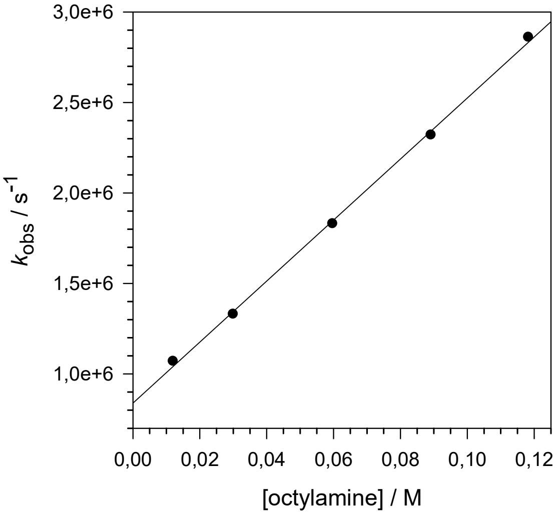

Figure S19. Plot of the observed rate constant (k_{obs}) against [substrate] for the reaction of octyl amine (**32**) with CumO$^{•}$ generated by 355 nm LFP of an Ar-saturated MeCN solution containing 1.0 M dicumyl peroxide measured at T = 25 °C following the decay of CumO$^{•}$ at 490 nm. From the linear regression analysis: intercept = 8.39 × 10^{5} s^{-1}, k_H = 1.69 × 10^{7} M^{-1} s^{-1}, r^{2} = 0.9988.

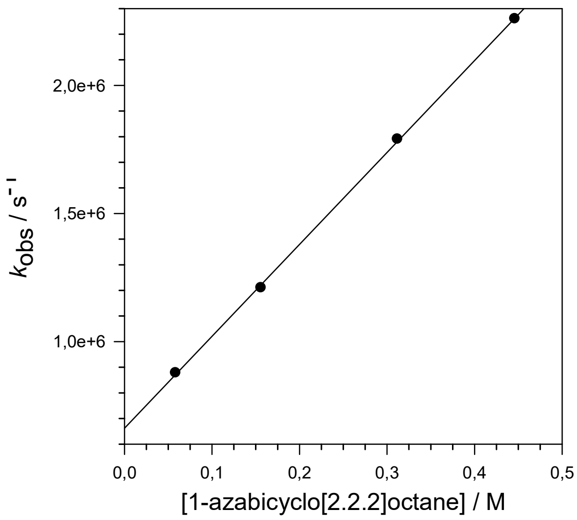

Figure S20. Plot of the observed rate constant (k_{obs}) against [substrate] for the reaction of 1-azabicyclo[2.2.2]octane (**46**) with CumO$^{•}$ generated by 355 nm LFP of an Ar-saturated MeCN solution containing 1.0 M dicumyl peroxide measured at T = 25 °C following the decay of CumO$^{•}$ at 490 nm. From the linear regression analysis: intercept = 6.62 × 10^{5} s^{-1}, k_H = 3.59 × 10^{6} M^{-1} s^{-1}, r^{2} = 0.9998.

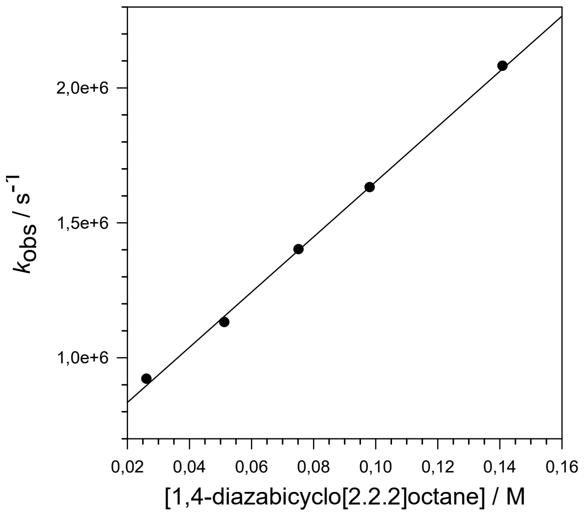

Figure S21. Plot of the observed rate constant (k_{obs}) against [substrate] for the reaction of 1,4-diazabicyclo[2.2.2]octane (**47**) with CumO• generated by 355 nm LFP of an Ar-saturated MeCN solution containing 1.0 M dicumyl peroxide measured at T = 25 °C following the decay of CumO• at 490 nm. From the linear regression analysis: intercept = 6.30×10^5 s^{-1}, $k_H = 1.02 \times 10^7$ M^{-1} s^{-1}, $r^2 = 0.9985$.

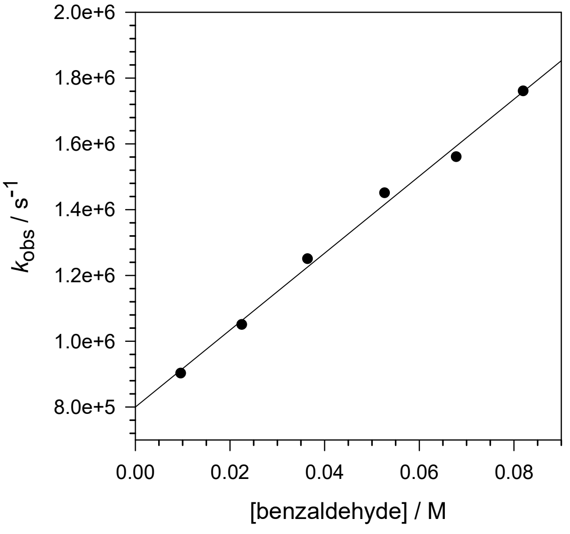

Figure S22. Plot of the observed rate constant (k_{obs}) against [substrate] for the reaction of benzaldehyde (**50**) with CumO• generated by 355 nm LFP of an Ar-saturated MeCN solution containing 1.0 M dicumyl peroxide measured at T = 25 °C following the decay of CumO• at 490 nm. From the linear regression analysis: intercept = 8.00×10^5 s^{-1}, $k_H = 1.17 \times 10^7$ M^{-1} s^{-1}, $r^2 = 0.9940$.

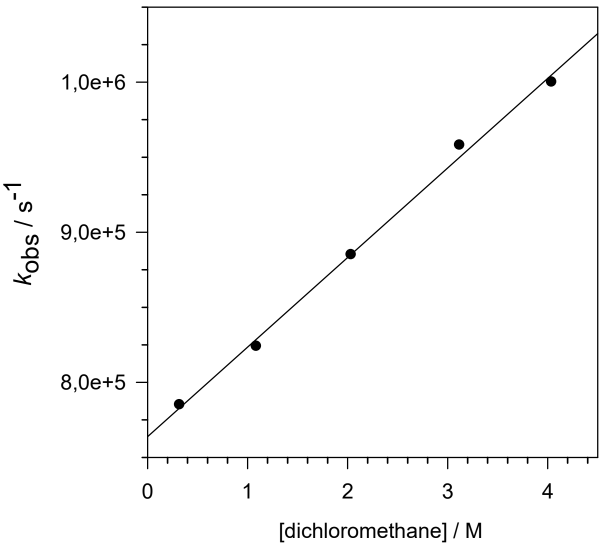

Figure S23. Plot of the observed rate constant (k_{obs}) against [substrate] for the reaction of dichloromethane (**52**) with CumO$^•$ generated by 355 nm LFP of an Ar-saturated MeCN solution containing 1.0 M dicumyl peroxide measured at T = 25 °C following the decay of CumO$^•$ at 490 nm. From the linear regression analysis: intercept = 7.64×10^5 s^{-1}, k_H = 5.97×10^4 M^{-1} s^{-1}, r^2 = 0.9964.

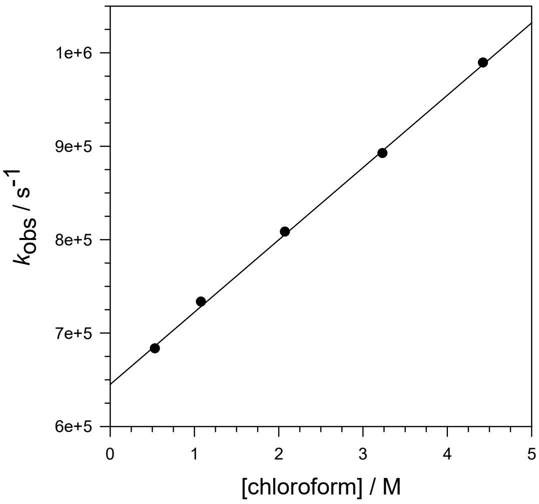

Figure S24. Plot of the observed rate constant (k_{obs}) against [substrate] for the reaction of chloroform (**53**) with CumO$^•$ generated by 355 nm LFP of an Ar-saturated MeCN solution containing 1.0 M dicumyl peroxide measured at T = 25 °C following the decay of CumO$^•$ at 490 nm. From the linear regression analysis: intercept = 6.45×10^5 s^{-1}, k_H = 7.74×10^4 M^{-1} s^{-1}, r^2 = 0.9992.

3) Table of k_H, k_H' and C–H BDE values employed for Figure 1

Table S1. Second-Order Rate Constants (k_H) for Reaction of the Cumyloxyl Radical (CumO•) with Hydrogen Atom Donor Substrates and BDE Values for the Pertinent C–H Bonds.

	**Substrate**	k_H [a] ($M^{-1}\ s^{-1}$)	k_H' [b] ($M^{-1}\ s^{-1}$)	**C–H BDE** [c] (kcal mol^{-1})
**aliphatic**				
**1**	$CH_3(CH_2)_3CH_3$	$3.1\pm0.3 \times 10^5$	5.2×10^4	99.15 [d]
**2**	$(H_3C)_3CCH_2CH_3$	$9.5\pm0.3 \times 10^4$	4.8×10^4	98.0
**3**	$(H_3C)_2CHCH(CH_3)_2$	$5.6\pm0.2 \times 10^5$	2.8×10^5	95.4
**4**	$(H_3C)_3CCH(CH_3)_2$	$2.2\pm0.2 \times 10^5$	2.2×10^5	*n.a.*
**5**	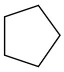	$9.54\pm0.08 \times 10^5$	9.54×10^4	95.6
**6**	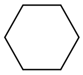	$1.1\pm0.1 \times 10^6$	9.2×10^4	99.5
**7**	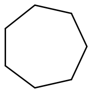	$2.20\pm0.02 \times 10^6$	1.57×10^5	94.0
**8**	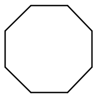	$3.2\pm0.1 \times 10^6$	2.00×10^5	95.7
**9**	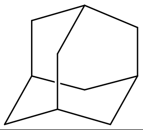	$6.90\pm0.07 \times 10^6$	(*tert*) 1.30×10^6<br>(*sec*) 1.37×10^5	96.2<br>98.4
**benzylic and allylic**				
**10**	$PhCH_3$	$1.85\pm0.08 \times 10^5$	6.3×10^4	89.7
**11**	$PhCH_2CH_3$	$7.9\pm0.1 \times 10^5$	4.0×10^5	85.4
**12**	$PhCH(CH_3)_2$	$5.6\pm0.3 \times 10^5$	5.6×10^5	83.2
**13**	$PhCH_2Ph$	$8.71\pm0.03 \times 10^5$	4.36×10^5	84.5
**14**	Ph_3CH	$3.04\pm0.08 \times 10^5$	3.04×10^5	81.0
**15**	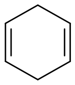	$6.56\pm0.03 \times 10^7$	1.64×10^7	76.0
**16**	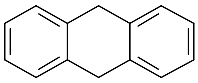	$5.04\pm0.01 \times 10^7$	1.26×10^7	76.3
**alcohols, ethers and diols**				
**17**	CH_3CH_2OH	$1.15\pm0.01 \times 10^6$	5.08×10^5	95.9
**18**	$CH_3CH_2CH_2OH$	$1.04\pm0.04 \times 10^6$	5.02×10^5	94.6 [e]
**19**	OH<br>| CH_3CHCH_3	$2.02\pm0.05 \times 10^6$	2.02×10^6	94.8
**20**	$HOCH_2CH_2OH$	$8.4\pm0.1 \times 10^5$	2.1×10^5	92.1

**21**	$HOCH_2CH_2CH_2OH$	$1.95\pm0.05 \times 10^6$	4.9×10^5	*n.a.*
**22**	$(CH_3CH_2)_2O$	$2.57\pm0.03 \times 10^6$	6.5×10^5	93.0
**23**	$PhCH_2OH$	$2.97\pm0.02 \times 10^6$	1.49×10^6	79.0
**24**	$PhCH_2OCH_2Ph$	$5.62\pm0.02 \times 10^6$	1.41×10^6	85.8
**25**	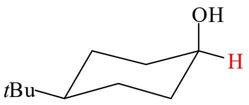	$5.06\pm0.03 \times 10^6$	5.06×10^6	*n.a.*
**26**	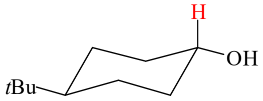	$2.37\pm0.02 \times 10^6$	2.37×10^6	*n.a.*
**27**	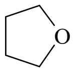	$5.8\pm0.1 \times 10^6$	1.45×10^6	92.1
**28**	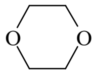	$8.2\pm0.2 \times 10^5$	1.03×10^5	96.5 [e]
**amines**				
**29**	$CH_3CH_2CH_2NH_2$	$1.10\pm0.02 \times 10^7$	5.05×10^6	91.0
**30**	$CH_3(CH_2)_3CH_2NH_2$	$1.55\pm0.02 \times 10^7$	7.75×10^6	90.5
**31**	$CH_3(CH_2)_4CH_2NH_2$	$1.68\pm0.02 \times 10^7$	8.4×10^6	90.5 [f]
**32**	$CH_3(CH_2)_6CH_2NH_2$	$1.69\pm0.02 \times 10^7$	8.45×10^6	90.5 [f]
**33**	$(CH_3CH_2CH_2)_2NH$	$1.01\pm0.03 \times 10^8$	2.53×10^7	90.0 [g]
**34**	$(CH_3CH_2CH_2)_3N$	$2.3\pm0.1 \times 10^8$	3.83×10^7	90.0
**35**	$(CH_3)_2CHCH_2NH_2$	$9.6\pm0.1 \times 10^6$	4.8×10^6	*n.a.*
**36**	$(CH_3CH_2)_3N$	$2.19\pm0.05 \times 10^8$	3.65×10^7	90.7
**37**	$PhCH_2NH_2$	$1.8\pm0.1 \times 10^7$	9.0×10^6	88.0
**38**	$(PhCH_2)_2NH$	$3.75\pm0.05 \times 10^7$	9.38×10^6	*n.a.*
**39**	$(CH_2{=}CHCH_2)_3N$	$6.15\pm0.06 \times 10^7$	1.03×10^7	82.6
**40**	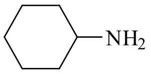	$2.1\pm0.1 \times 10^7$	2.1×10^7	94.6
**41**	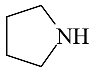	$1.24\pm0.05 \times 10^8$	3.1×10^7	88.6
**42**	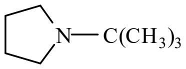	$3.0\pm0.1 \times 10^8$	7.5×10^7	*n.a.*
**43**	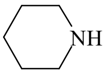	$1.07\pm0.01 \times 10^8$	2.68×10^7	91.2
**44**	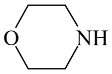	$5.0\pm0.2 \times 10^7$	1.25×10^7	92.0
**45**	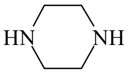	$2.26\pm0.01 \times 10^8$	2.83×10^7	91.5
**46**	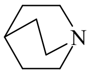	$3.5\pm0.2 \times 10^6$	5.8×10^5	96.2
**47**	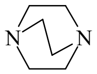	1.06×10^7	8.8×10^5	93.4

**other substrates**				
**48**	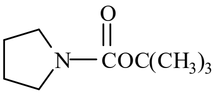	$1.4{\pm}0.1 \times 10^{7}$	3.5×10^{6}	*n.a.*
**49**	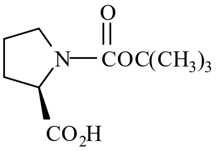	$2.51{\pm}0.08 \times 10^{6}$	1.26×10^{6}	*n.a.*
**50**	PhCHO	$1.23{\pm}0.05 \times 10^{7}$	1.23×10^{7}	88.7
**51**	$O{=}P[N(CH_3)_2]_3$	$1.87{\pm}0.02 \times 10^{7}$	1.04×10^{6}	*n.a.*
**52**	CH_2Cl_2	$6.0{\pm}0.2 \times 10^{4}$	3.0×10^{4}	95.7
**53**	$CHCl_3$	$7.7{\pm}0.4 \times 10^{4}$	7.7×10^{4}	93.8
**54**	$H_3C\overset{\overset{O}{\|}}{S}CH_3$	$1.8{\pm}0.1 \times 10^{4}$	3×10^{3}	94.0
**55**	$H_3C\overset{\overset{O}{\|}}{C}CH_3$	$< 1 \times 10^{4}$	$< 2 \times 10^{3}$	96.0
**56**	CH_3CN	$< 1 \times 10^{4}$	$< 3 \times 10^{3}$	97.0

[a]Measured in Ar or N_2-saturated MeCN solution at T = 25 °C by 355 nm LFP, for details see text. [b]$k_H' = k_H/n$, where n represents the number of equivalent abstractable hydrogen atoms. [c]Taken from ref. S2, recommended values (when available). [d]Average of the recommended values for the C–H bonds at C-2 and C-3 (99.2 and 99.1 kcal mol^{-1}, respectively). [e]Average of the given values. [f]Assumed to be identical to the available value for pentylamine (**30**). [g]Assumed to be identical to the available value for tripropylamine (**34**).

4) Product studies on the reaction of CumO• with adamantane

Product analysis of the reaction of CumO• with adamantane (**9**) has been carried out by 310 nm steady-state photolysis of an oxygen-saturated isooctane solution containing 0.1 M dicumyl peroxide and 0.4 M substrate. Under these conditions, HAT from the aliphatic C–H bonds of adamantane to CumO• occurs and the carbon-centered radicals thus formed are rapidly trapped by oxygen, with the resulting peroxyl radicals that evolve to give the oxygenated products. Since competitive HAT and β-cleavage pathways take place after CumO• generation,[S3] both cumyl alcohol and acetophenone are found in the reaction mixture along with the oxidation products.

The temperature was controlled at T = 25±0.5 °C by means of a thermostat linked to the outer casing of a Pyrex glass vessel containing the reaction mixture. A 5 ml solution of adamantane (272.5 mg, 0.4 M) and dicumyl peroxide (135.2 mg, 0.1 M) in isooctane is introduced in a jacketed Pyrex glass tube. The solution is saturated with oxygen and irradiated with 10×15W fluorescent lamps (emission maximum 310 nm) for 1 and 6 hours under magnetic stirring. The internal standard (4-*tert*-butylcyclohexanone) is then added and the solution is directly analyzed without any workup. GC analysis affords substrate conversion and product yields relative to the internal standard integration (Table S2). The reaction leads to the formation of 1-adamantanol, 2-adamantanone and 1,3-adamantanediol, identified by comparison with authentic samples.

Table S2. Reaction of the cumyloxyl radical (CumO•) with adamantane (**9**).

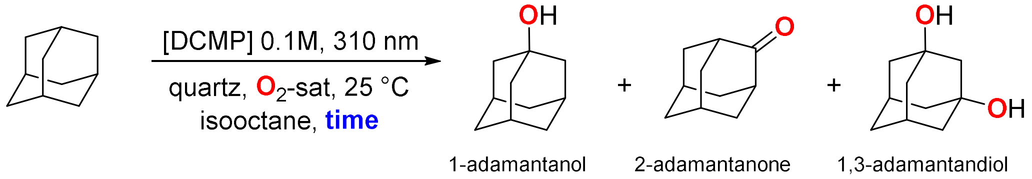

Time (h)	Conv (%)[a]	Product yield (%)[a]: 1-adamantanol[b]	2-adamantanone[b]	1,3-adamantandiol[b]	Total yield (%)
1	20.6	14.4	4.2	0.6	19.2
6	40.2	23.0	7.7	1.5	32.3

[a]Conversion and product yields are determined from the crude reaction mixture by GC analysis and are averaged over at least two independent experiments. [b]Identified by comparison with authentic samples.

5) Marcus analysis

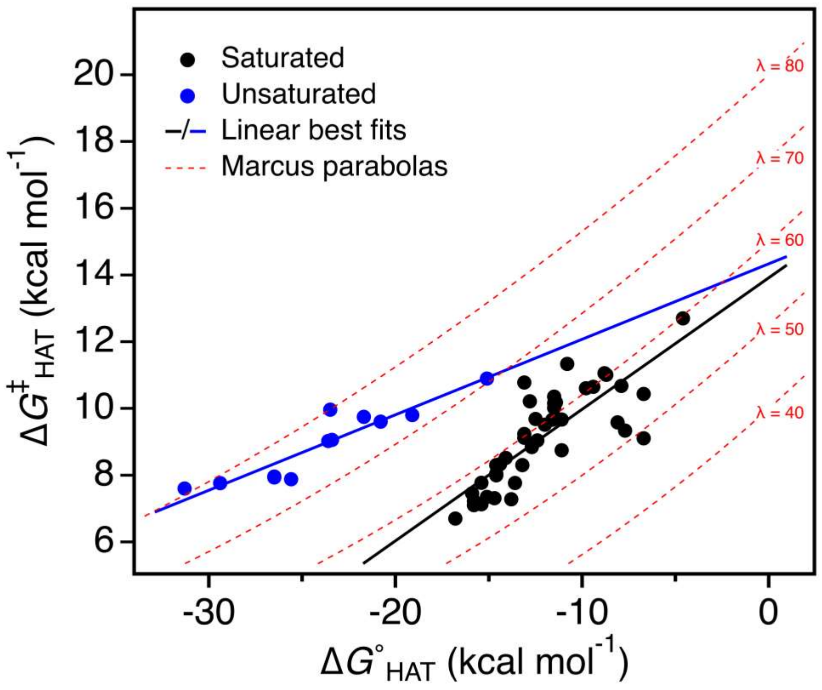

Figure S25. Plot of $\Delta G^{\ddagger}_{\mathrm{HAT}}$ vs $\Delta G^{\circ}_{\mathrm{HAT}}$ for reaction of cumyloxyl radical with saturated (black) and unsaturated (blue) substrates. $\Delta G^{\ddagger}_{\mathrm{HAT}}$ values were calculated from the normalized experimental rate constants k_{H}' using the Eyring equation, and the $\Delta G^{\circ}_{\mathrm{HAT}}$ values were calculated from the computed gas-phase BDFEs of cumyloxyl radical O–H and substrates C–H bonds (see main text). The use of gas-phase $\Delta G^{\circ}_{\mathrm{HAT}}$ values assumes that the difference in free energy to solvate RH and $R^{\bullet}$ is the same for substrate and radical (in fact, both differences are assumed to be negligible for small organic molecules). The solid lines are linear fits of the saturated and unsaturated data. The dashed lines are Marcus parabolas corresponding to selected values of λ, which highlight the poor agreement of the unsaturated data with this model.

6) Plots of ΔG^0_{HAT} *vs* IP and $\Delta G^{\neq}_{HAT}$ *vs* IP

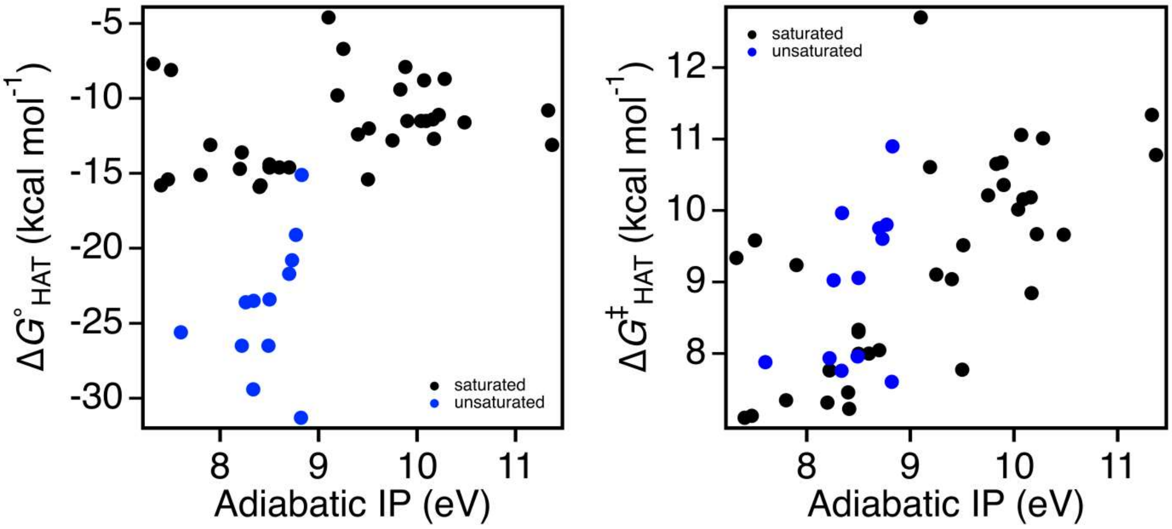

Figure S26. Plots of ΔG^0_{HAT} *vs* IP and $\Delta G^{\neq}_{HAT}$ *vs* IP for substrates **1-9**, **17-20**, **22**, **27-36**, **40**, **41**, **43**, **44**, **46**, **47**, **50-54** (saturated group) and **10-16**, **23**, **24**, **37-39** (unsaturated group). $\Delta G°_{HAT}$, is the computed BDFE for the C–H bond minus BDFE(CumO–H) (see text). $\Delta G^{\ddagger}_{HAT}$ is obtained from HAT rate constants displayed in Table 1 and Table 2 using the Eyring equation. Adiabatic IPs are taken from the NIST database (https://webbook.nist.gov/chemistry/).

7) Theoretical calculations

7.1) Benchmarking

The C–H BDEs for a variety of compounds were calculated with W1BD and compared to the data compiled by Luo (referred to as “Literature BDEs” in the foregoing)[S2] in Figure S27A, and to those obtained by ROCBS-QB3 in Figure S27B. The data are presented in graphical form below, and in tabular form in the Supporting Information. The set of BDEs includes species subjected to kinetic experiments, and was augmented by a number of small molecular species amenable to W1BD calculations.

Figure S27A shows that there is an excellent correlation between the W1BD calculated BDEs and those tabulated by Luo.[S2] The average absolute and signed difference between the calculated and tabulated values are 0.99 and -0.73 kcal/mol, respectively. There is some scatter in data about the best fit line, with absolute errors in some BDEs predicted to be in the 2-3 kcal/mol (n = 6) range for the set of 35 molecules. By comparison, Figure S27B shows that the errors in the set of 57 (RO)CBS-QB3 BDEs span a much larger range, but nevertheless have a high correlation coefficient: BDEs using this method are predicted to be in error by 4-5 kcal/mol (n = 2), 3-4 kcal/mol (n = 3), and 2-3 kcal/mol (n = 10). (RO)CBS-QB3 predicts BDEs with average absolute and signed differences relative to the tabulated values of 1.46 and -0.74 kcal/mol, respectively.

To assess the agreement between BDEs predicted by the W1BD and (RO)CBS-QB3 methods, we plotted the calculated values against each other in Figure S27C. The correlation is even higher than that shown in Fig. S27A, and there is much less scatter in the data. Indeed, the average absolute and signed differences between the two methods is only 0.31 and -0.08 kcal/mol, respectively, and the largest absolute deviations occurring for 1,4-cyclohexadiene, 1-3-pentadiene, and 1-4, pentadiene (1.3, 1.2, and 1.2 kcal/mol, respectively).

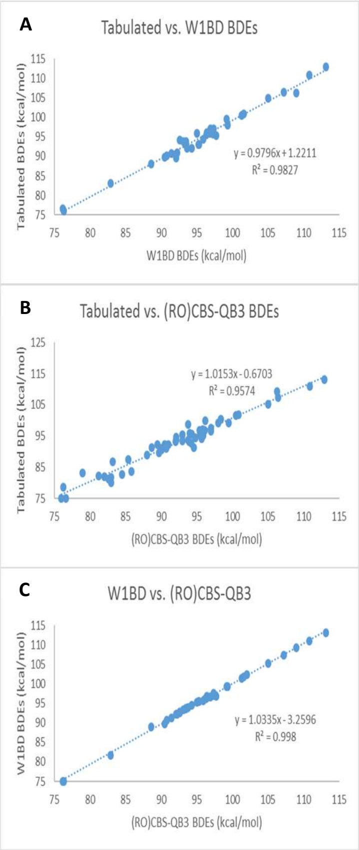

Figure S27. Tabulated versus A) W1BD calculated C–H BDEs for 35, and B) (RO)CBS-QB3 BDEs for 56 substrates. C) W1BD versus (RO)CBS-QB3 calculated BDEs for the species plotted in A).

W1BD is a composite computational method that attempts to predict atomic and molecular electronic energies at the fully correlated, complete basis set limit. A full description of the method is provided in ref. S4. In brief, the method utilizes B3LYP/cc-pVTZ+d for geometry optimization and frequency calculations. Subsequently, single-point energies are calculated using the Brueckner doubles with perturbative triples (BD(T)) approach with the augh-cc-pVXZ+2df (X=D,T) basis sets and BD calculations (without perturbative triples) are calculated with the augh-cc-pVQZ+2df basis set. These electronic energies are extrapolated to the complete basis set limit. BD(T) calculations are also performed using the MTSmall basis set to estimate scalar relativistic contributions to the energy via the Douglas-Kroll-Hess model. Barnes et al. showed in their work that W1BD predicts the atomization energies, electron affinities, ionization potentials, and proton affinities in the G2/97 data set with mean absolute errors of 0.55, 0.43, 0.35, and 0.43 kcal/mol, respectively, of the corresponding reference energies.[S4]

The results of our calculations suggest that some of the tabulated BDEs may require re-evaluation. More importantly for the present study, however, is that the results in Fig. 27C show that we are able to produce consistent, high-quality BDEs using (RO)CBS-QB3 across a wide range of systems and bond strengths. For comparisons to experimental rate constants, this consistency is required in order to allow us to make sound assessments as to the validity of the Bell-Evans-Polanyi Principle.

7.2) Marcus' inner-sphere reorganization energies

We estimated the self-exchange values, λ_i, following an approach based on Nelsen's 4-point method.[S5,S6] The summary of such calculations is shown in Table S3.

Table S3. Estimation of Marcus' inner-sphere reorganization energies for systems **1**, **10**, **15**, **19,** and **23**.

	Distortion Energies (kcal mol^{-1})			B3LYP/6-311G(2d,d,p) Energies (Hartree)			
**substrate**	**parent in radical.**[a]	**radical in parent.**[b]	**sum of distortion energies**	**parent**	**radical**	**distorted parent**	**distorted radical**
$CH_3(CH_2)_3CH_3$ **(1)**	6.8	17.7	24.5	-197.8291673	-197.1623151	-197.8009094	-197.1514328
$PhCH_3$ **(10)**	12.4	26.7	39.1	-271.6360424	-270.9839264	-271.5934525	-270.9641766
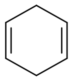 **(15)**	20.4	32.0	52.4	-233.4810867	-232.8544039	-233.430127	-232.8219136
OH CH_3CHCH_3 **(19)**	5.5	14.2	19.7	-194.4183444	-193.7618785	-194.3956533	-193.7531697
$PhCH_2OH$ **(23)**	17.8	40.7	58.5	-346.8682751	-346.2277859	-346.8034337	-346.1994483

[a]Single point energy for the optimized structure of the parent with the labile H atom removed minus the energy of the optimized radical. [b]Single point energy for the optimized structure of the radical with the labile H atom added minus the energy of the optimized parent. The coordinates of the added H were energy-optimized while the rest of the structure was kept frozen at the radical minimum.

7.3) Structures and thermochemical data

In a separate zip file, we have compiled all the optimized structures utilized to calculate the BDEs and BFDEs displayed in Table 3. The geometries are presented using the XYZ file format and are labeled as X_parent.xyz or X_radical.xyz, where X is the number of the system as depicted in Table 3. We also include the file “thermochemical_data.csv” which contains the E(0 K), ZPE, H298, and G298 terms, in Hartrees, for all systems.

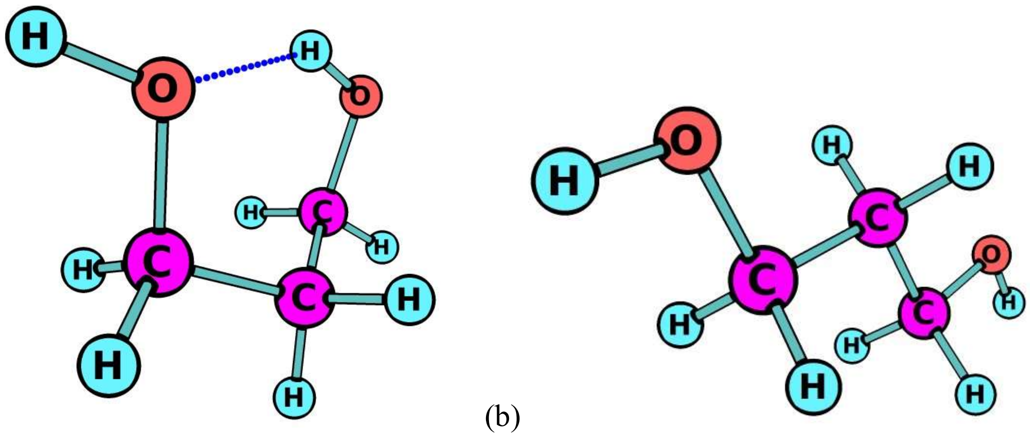

(a) (b)

Figure S28. Intramolecularly hydrogen bonded (a) and non-hydrogen bonded (b) conformations of 1,3-propanediol (**21**). The bonded structure is 3.5 kcal mol^{-1} more stable in acetonitrile than the non-bonded molecule. The BDEs for the C–H α to the hydrogen bond donor and acceptor are 94.0 and 96.0 kcal mol^{-1}, respectively. Analogous structures for 1,2-ethanediol (**21**) show a 2.5 kcal mol^{-1} preference for the bonded geometry with respect to the non-hydrogen bonded one in acetonitrile and BDEs of 95.1 and 97.4 kcal mol^{-1}, for the C–H α to the HB donor and acceptor, respectively.

8) References

(S1) Finn, M.; Friedline, R.; Suleman, N. K.; Wohl, C. J.; Tanko, J. M. Chemistry of the *t*-Butoxyl Radical: Evidence that Most Hydrogen Abstractions from Carbon are Entropy-Controlled. *J. Am. Chem. Soc.* **2004**, *126*, 7578-7584.

(S2) Luo, Y.-R. Comprehensive Handbook of Chemical Bond Energies; CRC Press: Boca Raton, FL, 2007.

(S3) Salamone, M.; Bietti, M. Reaction Pathways of Alkoxyl Radicals. The Role of Solvent Effects on C−C Bond Fragmentation and Hydrogen Atom Transfer Reactions. *Synlett* **2014**, *25*, 1803-1816.

(S4) Barnes, E. C.; Petersson, G. A.; Montgomery, Jr., J. A.; Frisch, M. J.; Martin, J. M. L. Unrestricted Coupled Cluster and Brueckner Doubles Variations of W1 Theory. *J. Chem. Theory Comput.* **2009**, *5*, 2687-2693.

(S5) Nelsen, S. F.; Blackstock, S. C.; Kim, Y. Estimation of Inner Shell Marcus Terms for Amino Nitrogen Compounds by Molecular Orbital Calculations. *J. Am. Chem. Soc.* **1987**, *109*, 677-682.

(S6) Auer, B.; Fernandez, L. E.; Hammes-Schiffer, S. Theoretical Analysis of Proton Relays in Electrochemical Proton-Coupled Electron Transfer. *J. Am. Chem. Soc.* **2011**, *133*, 8282-8292.
